# Supplementary material for: Activity-dependent neuromodulation and calcium homeostasis cooperate to produce robust and modulable neuronal function
Source: PLoS Comput Biol. 2026 Apr 15;22(4):e1014177. doi: 10.1371/journal.pcbi.1014177 (PMC13102308; doi:10.1371/journal.pcbi.1014177)
Supplement: S1 Appendix — We provide all the elements required to reproduce the results presented in this paper, as well as additional experiments and their results. This includes, for example, model equations and their parameter values. (PDF) [file pcbi.1014177.s001.pdf]

S1 Appendix

Activity-dependent neuromodulation and calcium homeostasis cooperate to produce robust and modulable neuronal function

We present all the elements needed in addition to the main text to reproduce the results reported in this paper. We also provide some additional experiments on another conductance-based model that is not central to get the main message of the paper. Code and data can be found in the first author GitHub (https://github.com/arthur-fyon/BIOCONTROL_2025).

A Simulation details for the two conductance-based models

Two high-dimensional conductance-based models are employed. The first is the Stomatogastric Ganglion (STG) neuron model proposed by Liu et al. [S1]. Its membrane potential equation is:

$$
\begin{aligned}
C\dot{V} = &- \bar{g}_{\mathrm{Na}} m_{\mathrm{Na}}^3 h_{\mathrm{Na}} (V - E_{\mathrm{Na}}) - \bar{g}_{\mathrm{CaT}} m_{\mathrm{CaT}}^3 h_{\mathrm{CaT}} (V - E_{\mathrm{Ca}}) - \bar{g}_{\mathrm{CaS}} m_{\mathrm{CaS}}^3 h_{\mathrm{CaS}} (V - E_{\mathrm{Ca}}) \\
&- \bar{g}_{\mathrm{A}} m_{\mathrm{A}}^3 h_{\mathrm{A}} (V - E_{\mathrm{K}}) - \bar{g}_{\mathrm{KCa}} m_{\mathrm{KCa}}^4 (V - E_{\mathrm{K}}) - \bar{g}_{\mathrm{Kd}} m_{\mathrm{Kd}}^4 (V - E_{\mathrm{K}}) \\
&- \bar{g}_{\mathrm{H}} m_{\mathrm{H}} (V - E_{\mathrm{H}}) - g_{\mathrm{leak}} (V - E_{\mathrm{leak}}) + I_{\mathrm{app}},
\end{aligned} \tag{S1}
$$

where the dot notation indicates time derivatives. Na stands for the sodium current, CaT for the T-type calcium current, CaS for the slow calcium current, A for the A-type potassium current, KCa for the calcium-controlled potassium current, Kd for the delayed rectified potassium current, H for the H current, and leak for the leakage current. The parameters $\bar{g}_i$ denote maximum ion channel conductances (in mS/cm^2), and E_i denote the fixed Nernst

reversal potentials. Activation and inactivation gating variables (m and h) are dimensionless quantities between 0 and 1, each governed by a first-order equation:

$$\tau_X(V)\frac{dX}{dt} = X_\infty(V) - X, \tag{S2}$$

where $X_\infty(V)$ is the steady-state gating value and $\tau_X(V)$ the voltage dependent time constant. Table S3 lists all steady-state functions, time constants, and gating exponents. The KCa activation variable additionally depends on intracellular calcium concentration.

Calcium dynamics follow:

$$\tau_{\mathrm{Ca}}\frac{d\mathrm{Ca}}{dt} = -\alpha_{\mathrm{Ca}}\left(I_{\mathrm{CaS}} + I_{\mathrm{CaT}}\right) - \mathrm{Ca} + \beta_{\mathrm{Ca}}.$$

Fixed parameters for the STG model are listed in Table S1. All STG voltage traces in this article are obtained by numerically integrating equation (S1) and its associated gating equations using the Julia language [S2].

Table S1. Fixed parameters for the STG model. Reversal potentials, calcium time constant, and calcium dynamics parameters used in the STG model.

E_{leak}	E_{Na}	E_{K}	E_{H}	E_{Ca}	τ_{Ca}	α_{Ca}	β_{Ca}
$-50\,\mathrm{mV}$	$50\,\mathrm{mV}$	$-80\,\mathrm{mV}$	$-20\,\mathrm{mV}$	$80\,\mathrm{mV}$	$20\,\mathrm{ms}$	$0.94\,\mathrm{mM\,nF\,nA^{-1}}$	$0.05\,\mathrm{\mu M}$

The second model is a Dopaminergic (DA) neuron model adapted from Qian et al. [S3], where SK channels are removed to facilitate bursting. Its membrane equation is:

$$\begin{aligned} C\dot{V} = &- \bar{g}_{\mathrm{Na}} m_{\mathrm{Na}}^3 h_{\mathrm{Na}}(V - E_{\mathrm{Na}}) - \bar{g}_{\mathrm{Kd}} m_{\mathrm{Kd}}^3 (V - E_{\mathrm{K}}) - \bar{g}_{\mathrm{CaL}} m_{\mathrm{CaL}}^2 (V - E_{\mathrm{Ca}}) \\ &- \bar{g}_{\mathrm{CaN}} m_{\mathrm{CaN}} (V - E_{\mathrm{Ca}}) - \bar{g}_{\mathrm{ERG}} m_{\mathrm{ERG}} (V - E_{\mathrm{K}}) - g_{\mathrm{leak}}(V - E_{\mathrm{leak}}) \\ &- \bar{g}_{\mathrm{NMDA}} \frac{(V - E_{\mathrm{NMDA}})}{1 + Mg \cdot \exp(-0.08V)/10} + I_{\mathrm{app}}, \end{aligned} \tag{S3}$$

where CaL represents the L-type calcium current, CaN the N-type calcium current, ERG the ERG current, NMDA the NMDA current, and Mg the extracellular magnesium concentration.

As in the STG model, gating variables follow first-order kinetics, except for the ERG and NMDA currents. Table S4 lists all steady-state functions, time constants, and gating exponents.

For the ERG current, the activation and inactivation variables o_{ERG} and i_{ERG} satisfy:

$$\frac{do_{\mathrm{ERG}}}{dt} = a_0(V) \cdot (1 - o_{\mathrm{ERG}} - i_{\mathrm{ERG}}) + b_i(V) \cdot i_{\mathrm{ERG}} - o_{\mathrm{ERG}} \cdot (a_i(V) + b_0(V)),$$

$$\frac{di_{\mathrm{ERG}}}{dt} = a_i(V) \cdot o_{\mathrm{ERG}} - b_i(V) \cdot i_{\mathrm{ERG}}.$$

The steady-state ERG gating variables are:

$$o_{\mathrm{ERG},\infty}(V) = \frac{a_0(V) \cdot b_i(V)}{a_0(V) \cdot (a_i(V) + b_i(V)) + b_0(V) \cdot b_i(V)},$$

$$i_{\mathrm{ERG},\infty}(V) = \frac{a_0(V) \cdot a_i(V)}{a_0(V)(a_i(V) + b_i(V)) + b_0(V) \cdot b_i(V)}.$$

Calcium dynamics follow:

$$\tau_{\mathrm{Ca}} \frac{d\mathrm{Ca}}{dt} = -\alpha_{\mathrm{Ca}} \left(I_{\mathrm{CaL}} + I_{\mathrm{CaN}} \right) - \mathrm{Ca} + \beta_{\mathrm{Ca}}.$$

with fixed parameters identical to those in Table S1. Reversal potentials and magnesium concentration for the DA model are given in Table S2.

Table S2. Fixed parameters for the DA model. Reversal potentials and magnesium concentration used in the DA model.

E_{Na}	E_{K}	E_{Ca}	E_{leak}	E_{NMDA}	Mg
60 mV	−85 mV	60 mV	−50 mV	0 mV	1.4

All DA voltage traces in this article are obtained by numerical integration of equation (S3) and its associated gating equations using Julia [S2]. Although the NMDA current appears in the model, it is not discussed in the main text; it is included here with a constant maximum conductance scaled relative to the leakage current.

Table S3. Gating functions and kinetics for the STG model. Steady-state activation/inactivation functions, time constants, and exponents for each ionic current in the STG model. All f functions refer to the generalized sigmoid function (Eq. S4).

Current I_i	p_i	q_i	$m_{i,\infty}(V)$ or $m_{i,\infty}(V,\mathrm{Ca})$	$h_{i,\infty}(V)$	$\tau_{m_i}(V)$	$\tau_{h_i}(V)$
I_{Na}	3	1	$f(V,0,1,-5.29,25.5)$	$f(V,0,1,5.18,48.9)$	$f(V,1.32,-1.26,-25,120)$	$f(V,0,0.67,-10,62.9)\cdot f(V,1.5,1,3.6,34.9)$
I_{Kd}	4	0	$f(V,0,1,-11.8,12.3)$	—	$f(V,7.2,-6.4,-19.2,28.3)$	—
I_{CaT}	3	1	$f(V,0,1,-7.2,27.1)$	$f(V,0,1,5.5,32.1)$	$f(V,21.7,-21.3,-20.5,68.1)$	$f(V,105,-89.8,-16.9,55)$
I_{CaS}	3	1	$f(V,0,1,-8.1,33)$	$f(V,0,1,6.2,60)$	$1.4+\frac{7}{\exp\left(\frac{V+27}{10}\right)+\exp\left(\frac{V+70}{-13}\right)}$	$60+\frac{150}{\exp\left(\frac{V+55}{9}\right)+\exp\left(\frac{V+65}{-16}\right)}$
I_{KCa}	4	0	$\frac{\mathrm{Ca}}{\mathrm{Ca}+3}\cdot f(V,0,1,-12.6,28.3)$	—	$f(V,90.3,-75.1,-22.7,46)$	—
I_{A}	3	1	$f(V,0,1,-8.7,27.2)$	$f(V,0,1,4.9,56.9)$	$f(V,11.6,-10.4,-15.2,32.9)$	$f(V,38.6,-29.2,-26.5,38.9)$
I_{H}	1	0	$f(V,0,1,6,70)$	—	$f(V,272,1499,-8.73,42.2)$	—
I_{leak}	0	0	—	—	—	—

Table S4. Gating functions and kinetics for the DA model. All f functions refer to the generalized sigmoid function (Eq. S4). Additionally, there is a current I_{ERG} described by the ERG channel, whose rate functions are defined as exponentials of the membrane potential V. The activation and inactivation parameters are as follows: $a_0(V) = 0.0036\exp(0.0759V)$, $b_0(V) = 1.2523\times10^{-5}\exp(-0.0671V)$, $a_i(V) = 0.1\exp(0.1189V)$, and $b_i(V) = 0.003\exp(-0.0733V)$.

Current	p_i	q_i	$m_{i,\infty}(V)$ or $m_{i,\infty}(V,\mathrm{Mg})$	$h_{i,\infty}(V)$	$\tau_{m_i}(V)$	$\tau_{h_i}(V)$
I_{Na}	3	1	$f(V,0,1,-9.7264,30.0907)$	$f(V,0,1,10.7665,54.0289)$	$0.01+\frac{1.0}{(-\frac{15.6504+0.4043V}{\exp(-19.565-0.5052V)-1.0})+3.0212\exp(-0.007463V)}$	$0.4+\frac{1.0}{(0.00050754\exp(-0.063213V))+9.7529\exp(0.13442V)}$
I_{Kd}	3	0	$f(V,0,1,-12,25)$	—	$f(V,20,-18,-10,38)$	—
I_{CaL}	2	0	$f(V,0,1,-2,50)$	—	$f(V,30,-28,-3,45)$	—
I_{CaN}	1	0	$f(V,0,1,-7,30)$	—	$f(V,30,-25,-6,55)$	—
I_{NMDA}	1	0	$\frac{1}{1+\frac{\mathrm{Mg}\cdot\exp(-0.08V)}{10}}$	—	—	—
I_{leak}	0	0	—	—	—	—

$$f(V,A,B,C,D) = A + \frac{B}{1+\exp\left(\frac{V+D}{C}\right)} \tag{S4}$$

B Computation details for the neuromodulation controller

The neuromodulation controller [S4] employs the concept of Dynamic Input Conductances (DICs) introduced in Drion et al. [S5]. DICs consist of three voltage-dependent conductances that separate according to timescales: one fast, one slow, and one ultraslow. These DICs have been demonstrated to shape neuronal spiking. Specifically, based on specific values of the DICs, it becomes possible to predict the firing pattern of the neuron. The computation of the DICs in this article follows the improvements of [S6].

The DICs are three voltage-dependent conductances, denoted as $g_{\mathrm{f}}(V)$, $g_{\mathrm{s}}(V)$, and $g_{\mathrm{u}}(V)$, which can be computed as linear functions of the maximal conductance vector $\bar{g}_{\mathrm{ion}} \in \mathbb{R}^N$ of an N-channel conductance-based model at each voltage level V

$$\begin{bmatrix} g_{\mathrm{f}}(V) \\ g_{\mathrm{s}}(V) \\ g_{\mathrm{u}}(V) \end{bmatrix} = f_{\mathrm{DIC}}(V) = S(V) \cdot \bar{g}_{\mathrm{ion}} \,, \tag{S5}$$

where $S(V) \in \mathbb{R}^{3\times N}$ is a sensitivity matrix that can be built by (line per line)

$$S_{\mathrm{f}\,j}(V) = -\left(w_{\mathrm{fs},X_j} \cdot \frac{\partial \dot{V}}{\partial X_j} \frac{\partial X_{j,\infty}}{\partial V} \right) \Big/ g_{\mathrm{leak}}, \tag{S6}$$

$$S_{\mathrm{s}\,j}(V) = -\left((w_{\mathrm{su},X_j} - w_{\mathrm{fs},X_j}) \cdot \frac{\partial \dot{V}}{\partial X_j} \frac{\partial X_{j,\infty}}{\partial V} \right) \Big/ g_{\mathrm{leak}}, \tag{S7}$$

$$S_{\mathrm{u}\,j}(V) = -\left((1 - w_{\mathrm{su},X_j}) \cdot \frac{\partial \dot{V}}{\partial X_j} \frac{\partial X_{j,\infty}}{\partial V} \right) \Big/ g_{\mathrm{leak}}, \text{ with } j = 1:N, \tag{S8}$$

where equations (S6), (S7), and (S8) represent the computation of rows 1, 2, and 3, respectively, of the matrix $S(V)$ — that is, the computation of the fast, slow, and ultraslow DICs. The weighing factors w_{fs,X_j} and w_{su,X_j} determine how each variable contributes to different timescales. Their values are computed based on a logarithmic scaling

between 0 and 1, using reference timescales:

$$w_{\mathrm{fs},X_j}(V) = \begin{cases} 1 & , \quad \tau_{X_j}(V) \le \tau_{\mathrm{f}}(V), \\ \dfrac{\log(\tau_{\mathrm{s}}(V)) - \log\left(\tau_{X_j}(V)\right)}{\log(\tau_{\mathrm{s}}(V)) - \log(\tau_{\mathrm{f}}(V))} & , \quad \tau_{\mathrm{f}}(V) < \tau_{X_j}(V) \le \tau_{\mathrm{s}}(V), \\ 0 & , \quad \tau_{X_j}(V) > \tau_{\mathrm{s}}(V), \end{cases}$$

$$w_{\mathrm{su},X_j}(V) = \begin{cases} 1 & , \quad \tau_{X_j}(V) \le \tau_{\mathrm{s}}(V), \\ \dfrac{\log(\tau_{\mathrm{u}}(V)) - \log\left(\tau_{X_j}(V)\right)}{\log(\tau_{\mathrm{u}}(V)) - \log(\tau_{\mathrm{s}}(V))} & , \quad \tau_{\mathrm{s}}(V) < \tau_{X_j}(V) \le \tau_{\mathrm{u}}(V), \\ 0 & , \quad \tau_{X_j}(V) > \tau_{\mathrm{u}}(V). \end{cases} \tag{S9}$$

The reference timescales τ_{f}, τ_{s}, and τ_{u} are chosen according to the characteristic timescales of bursting and spiking dynamics [S5]. τ_{f} corresponds to the activation time constant of the fastest depolarizing current, while τ_{s} represents the activation time constant of the fastest repolarizing current. Finally, τ_{u} is associated with the slowest variable in the system, which typically governs burst adaptation.

X_j corresponds to the gating variable(s) (activation and/or inactivation) of the current with index j, and $X_{j,\infty}$ denotes the steady-state function(s) of the considered gating variable lag equation(s). It is important to note that if the current j has both activation and inactivation variables, the column S_j corresponds to the sum of equations (S6) through (S8) over the two gating variables of the current j.

While the complete curve of the DICs may be of interest, only its value at the threshold voltage V_{th} is utilized, as the values and signs of the DICs at V_{th} reliably determine the neuronal firing pattern [S5]. In the following sections, the voltage dependency of all variables will be disregarded, as functions are only evaluated at V_{th}. The threshold voltage is computed as $g_{\mathrm{in}}(V_{\mathrm{th}}) = g_{\mathrm{f}}(V_{\mathrm{th}}) + g_{\mathrm{s}}(V_{\mathrm{th}}) + g_{\mathrm{u}}(V_{\mathrm{th}}) < 0$, while ensuring that $g_{\mathrm{in}}(V_{\mathrm{th}} - \delta V) \ge 0$ with any arbitrarily small $\delta V > 0$. It is important to note that this algorithm might fail for the DA neuron model, where the default $V_{\mathrm{th}} = -55.5$ mV.

The neuromodulation controller uses user-defined target values of DICs and dynamically drives the modulated conductances to converge to them. More formally, if the neuron expresses N types of ions, of which n are modulated and $m = N - n$ are unmodulated, the complete sensitivity matrix S used in the computation of the DICs at threshold voltage and the complete maximum ion channel conductance vector $\bar{g}_{\mathrm{ion}}$ can be split in modulated ($S_{\mathrm{mod}} \in \mathbb{R}^{p \times n}$, $\bar{g}_{\mathrm{mod}} \in \mathbb{R}^{n}$) and unmodulated ($S_{\mathrm{unmod}} \in \mathbb{R}^{p \times m}$, $\bar{g}_{\mathrm{unmod}} \in \mathbb{R}^{m}$) components. Then the mapping from

maximal conductances to DICs at threshold voltage can then be written as

$$f_{\mathrm{DIC}} = \begin{bmatrix} S_{\mathrm{mod}} & S_{\mathrm{unmod}} \end{bmatrix} \cdot \begin{bmatrix} \bar{g}_{\mathrm{mod}} \\ \bar{g}_{\mathrm{unmod}} \end{bmatrix} . \tag{S10}$$

Isolating the modulated ion channel references as unknowns, the system becomes

$$S_{\mathrm{mod}} \cdot \bar{g}_{\mathrm{mod}} = f_{\mathrm{DIC}} - S_{\mathrm{unmod}} \cdot \bar{g}_{\mathrm{unmod}} =: f_{\mathrm{DIC_r}} . \tag{S11}$$

Under the assumption that $n \geq p$ and S_{mod} has full row rank, the solution set to (S11) is

$$\{ \ \bar{g}_{\mathrm{mod}} \mid S_{\mathrm{mod}} \cdot \bar{g}_{\mathrm{mod}} = f_{\mathrm{DIC_r}} \ \} = \{ \ \bar{g}_0 + z \mid z \in \mathcal{N}(S_{\mathrm{mod}}) \ \} , \tag{S12}$$

where $\bar{g}_0$ is any solution, *i.e.*, $S_{\mathrm{mod}} \cdot \bar{g}_0 = f_{\mathrm{DIC_r}}$ and $\mathcal{N}(S_{\mathrm{mod}})$ is the nullspace of S_{mod}, *i.e.*,

$$\mathcal{N}(S_{\mathrm{mod}}) = \{z \in \mathbb{R}^n \mid S_{\mathrm{mod}} z = 0\}. \tag{S13}$$

An important particular solution that has the smallest norm as compared to any other solution, is

$$\bar{g}_0 = S_{\mathrm{mod}}^T (S_{\mathrm{mod}} S_{\mathrm{mod}}^T)^{-1} \cdot f_{\mathrm{DIC_r}} =: S_{\mathrm{mod}}^+ \cdot f_{\mathrm{DIC_r}} , \tag{S14}$$

where S_{mod}^+ is the Moore-Penrose generalized inverse of S_{mod} [S7]. The matrix

$$S_{\mathrm{mod}}^+ S_{\mathrm{mod}} = P_{\mathcal{N}(S_{\mathrm{mod}})^\perp} \tag{S15}$$

gives the projection onto the orthogonal complement of $\mathcal{N}(S_{\mathrm{mod}})$. Equation S14 provides reference values $\bar{g}_0$ for modulated ion channel expressions to the neuron to the desired excitability type. Biologically, a molecular regulatory network can implement this equation.

The last part of the controller consists of a two-compartment model containing modulated conductances, controlled by a classical PI controller that dynamically drives $\bar{g}_{\mathrm{mod}}$ to track $\bar{g}_0$. In all experiments, only the slow and ultraslow DICs were tuned, as these are required to trigger transitions between tonic firing and bursting [S4, 6]. For the STG model, the modulated conductances are $\bar{g}_{\mathrm{CaS}}$ and $\bar{g}_{\mathrm{A}}$, while for the DA model, they are $\bar{g}_{\mathrm{CaL}}$ and $\bar{g}_{\mathrm{CaN}}$.

C Computation details for the homeostatic controller

The homeostatic controller from [S8] integrates the calcium error between the actual and target values and uses a proportional-integral action to tune all model conductances so that the mean calcium level converges to the target. Formally, for each conductance $\forall i \in [1, N]$, the controller is defined by:

$$\tau_i \dot{m}_i = [Ca^{+2}]_{\text{target}} - [Ca^{+2}],$$
$$\tau_g \dot{\bar{g}}_i = m_i - \bar{g}_i,$$

where $[Ca^{2+}]$ and $[Ca^{2+}]_{\text{target}}$ represent the current and target intracellular calcium concentrations, respectively. The parameter τ_i denotes the transcription time constant of conductance i, while τ_g is a shared translation time constant across all conductances. The variable m_i corresponds to the mRNA level of channel i.

In essence, the calcium error is integrated into the mRNA levels, which in turn modulate the associated channel conductances. This regulation drives the calcium concentration toward its target, as larger channel conductances—particularly calcium-conducting ones—enable greater calcium influx. Starting from any initial condition, the controller guides the system along a trajectory in conductance space known as the homogeneous scaling line, which passes through the origin and the initial condition. At steady state, the conductance ratios remain fixed, such that:

$$\frac{\bar{g}_i}{\bar{g}_j} = \frac{\tau_j}{\tau_i}. \tag{S16}$$

This invariance preserves the relative scaling of conductances during homeostatic compensation. As a result, the system evolves along the homogeneous scaling beam defined by these ratios.

In all experiments, the calcium target was set to $[Ca^{2+}]_{\text{target}} = 125\,\mu\text{M}$ for the STG model and $[Ca^{2+}]_{\text{target}} = 3\,\mu\text{M}$ for the DA model. To allow both controllers to operate on comparable timescales, the homeostatic controller was substantially accelerated relative to its original formulation by setting $\tau_g = 100\,\text{ms}$ in both models. The transcription time constant for sodium was fixed at $\tau_{\text{Na}} = 600\,\text{ms}$, and the remaining transcription constants were initialized using:

$$\tau_i = \tau_{\text{Na}} \cdot \frac{\bar{g}_{\text{Na}}}{\bar{g}_i}, \tag{S17}$$

where the conductance values are those at the beginning of the simulation. These constants were not recomputed once neuromodulation was applied.

D Computation details for Figs 2 and 3

For both models, the initial degenerate populations were taken from [S6], which introduces an efficient method for generating degenerate neuronal populations using DICs. For spiking behavior, the DIC pairs were set to $(g_\mathrm{s}, g_\mathrm{u}) = (5, 4)$ for the STG model and $(0.5, 5)$ for the DA model. For bursting behavior, they were set to $(g_\mathrm{s}, g_\mathrm{u}) = (-8, 4)$ for the STG model and $(-4, 5)$ for the DA model [S6].

In the case of sharp neuromodulation, only the homeostatic controller acts on the conductances. Midway through the simulation, g_mod was instantaneously set to the corresponding $\bar{g}_0$ value without any form of dynamic control. After the transition from tonic firing to bursting occurred, the homeostatic controller remained active to reduce intracellular calcium.

In contrast, in the case of controlled neuromodulation, both controllers operated throughout the simulation.

E Computation details for Fig 5

Blockade of a channel was modeled by setting its conductance to $\bar{g}_i = 0$. However, such blockades may require adjusting the pair $(g_\mathrm{s}, g_\mathrm{u})$ to achieve maximal robustness in compensation. For the H and KCa channel blockades, $(g_\mathrm{s}, g_\mathrm{u}) = (-8, -4)$ was used, whereas for the Na and CaT channel blockades, the default pair $(g_\mathrm{s}, g_\mathrm{u}) = (-8, 4)$ was retained.

F Computation details for Fig 6

To achieve slower bursting in both green and yellow neurons (resembling the gastric mill rhythm), the time constant of the KCa channels was multiplied by 20.

The inhibitory synaptic current was modeled as:

$$I_\mathrm{syn} = \bar{g}_\mathrm{syn} \cdot m_\mathrm{syn}(V_\mathrm{pre}, t) \cdot (V_\mathrm{post} - E_\mathrm{syn}), \tag{S18}$$

where the subscripts pre and post refer to the pre- and postsynaptic neurons, respectively, and syn denotes synaptic.

Here, I_{syn} is the synaptic current added to the voltage equation of the postsynaptic neuron, $\bar{g}_{\text{syn}} = 0.8\,\text{mS/cm}^2$ is the fixed maximum synaptic conductance, m_{syn} is the synaptic gating variable following the dynamics from [S9], and $E_{\text{syn}} = -75\,\text{mV}$ is the inhibitory reversal potential.

The Ohmic (gap junction-like) connection current was modeled as:

$$I_\Omega = \bar{g}_\Omega \cdot (V_{\text{post}} - V_{\text{pre}}), \tag{S19}$$

where $\bar{g}_\Omega = 0.05\,\text{mS/cm}^2$ is the coupling conductance.

G Single-neuron conductance trajectories for Fig 2

To complement the population-level results shown in Figs 2 and 3 of the main text, we present detailed conductance trajectories for three representative neurons selected from the degenerate population of $N = 200$ STG neuron models. These neurons were chosen to illustrate the range of outcomes observed across the population.

For sharp neuromodulation (Fig S1), panels A, C, and E show the time evolution of all conductances on a logarithmic scale for each of the three neurons, corresponding to the population view in Fig 2A of the main text. Panels B, D, and F show the corresponding trajectories in the modulated conductance plane ($\bar{g}_{\text{CaS}}$, $\bar{g}_{\text{A}}$), with color indicating the intracellular calcium level along the trajectory, as in Fig 3 of the main text. Despite similar initial firing patterns, the three neurons follow distinct paths during calcium-homeostatic compensation, leading to heterogeneous outcomes: some neurons maintain bursting with altered properties, while others lose bursting entirely, consistent with the population-level results in Fig 2C.

For controlled neuromodulation (Fig S2), panels A, C, and E show the analogous conductance time evolution for the same three neurons, corresponding to Fig 2D of the main text. Panels B, D, and F show the trajectories in the modulated conductance plane with calcium-colored paths. In contrast to the sharp neuromodulation case, all three neurons converge to stable bursting states with appropriate intracellular calcium levels. The trajectories in the modulated conductance plane confirm that controlled neuromodulation maintains each neuron on a bursting-compatible scaling direction during calcium-homeostatic compensation, as described in Fig 3D and Fig 4B of the main text.

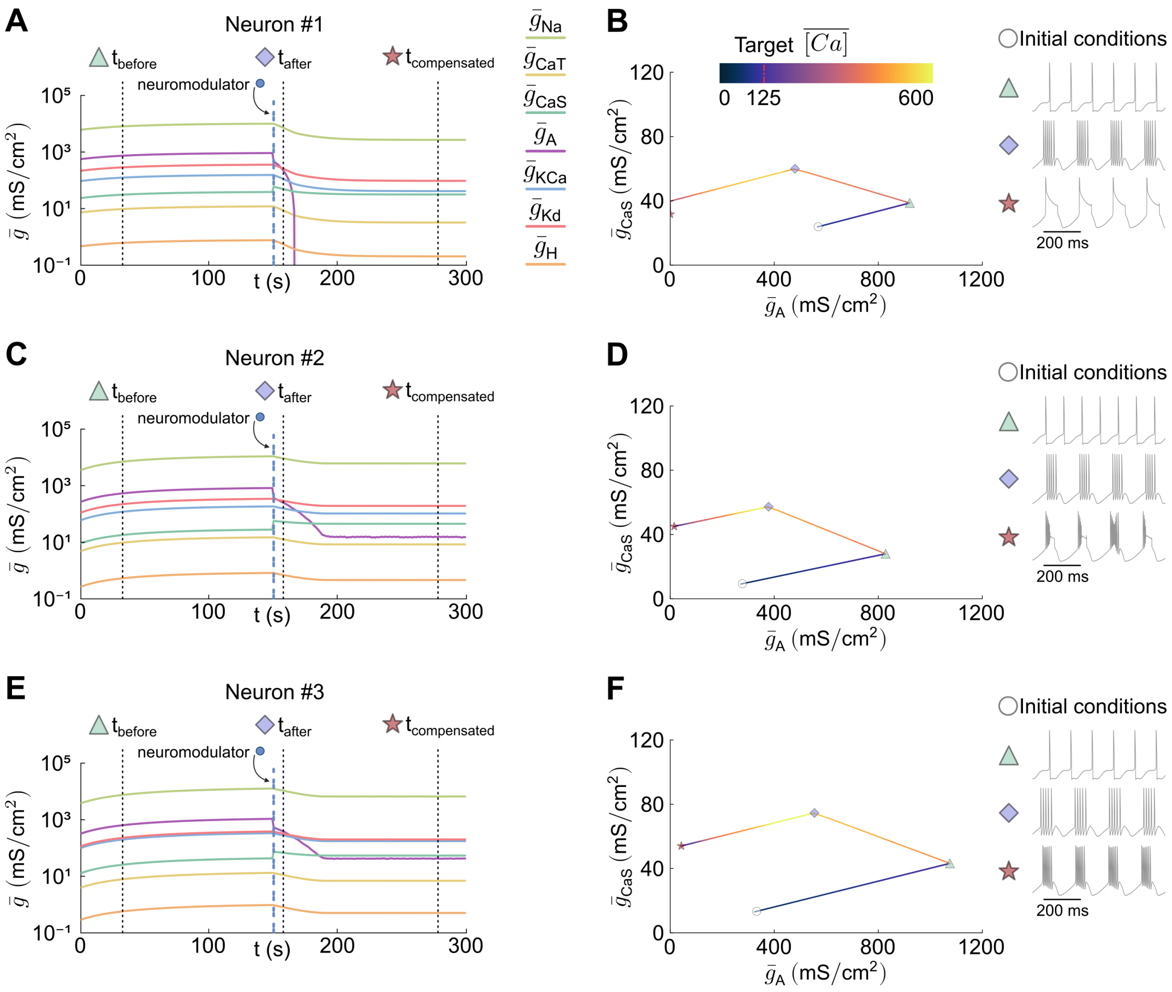

Fig S1. Single-neuron conductance trajectories under sharp neuromodulation with calcium homeostasis. Three representative neurons from the degenerate population of Fig 2A–C are shown. **A, C, E.** Time evolution of all conductances on a logarithmic scale for each neuron. **B, D, F.** Corresponding trajectories in the modulated conductance plane ($\bar{g}_{\mathrm{CaS}}$, $\bar{g}_{\mathrm{A}}$), with color encoding the intracellular calcium concentration along the trajectory. Each row corresponds to one neuron.

H Controlled neuromodulation and calcium homeostasis in the DA model

To demonstrate the generality of the framework beyond STG neurons, we applied the same combination of controlled neuromodulation and calcium homeostasis to a midbrain dopaminergic (DA) neuron model adapted from [S3], as discussed in the main text. The modulated conductances are $\bar{g}_{\mathrm{CaN}}$ and $\bar{g}_{\mathrm{CaL}}$, and the DIC pairs are as specified in section E.

The results are shown in Fig S3, which follows the same layout as Fig 2D–F of the main text. As in the STG

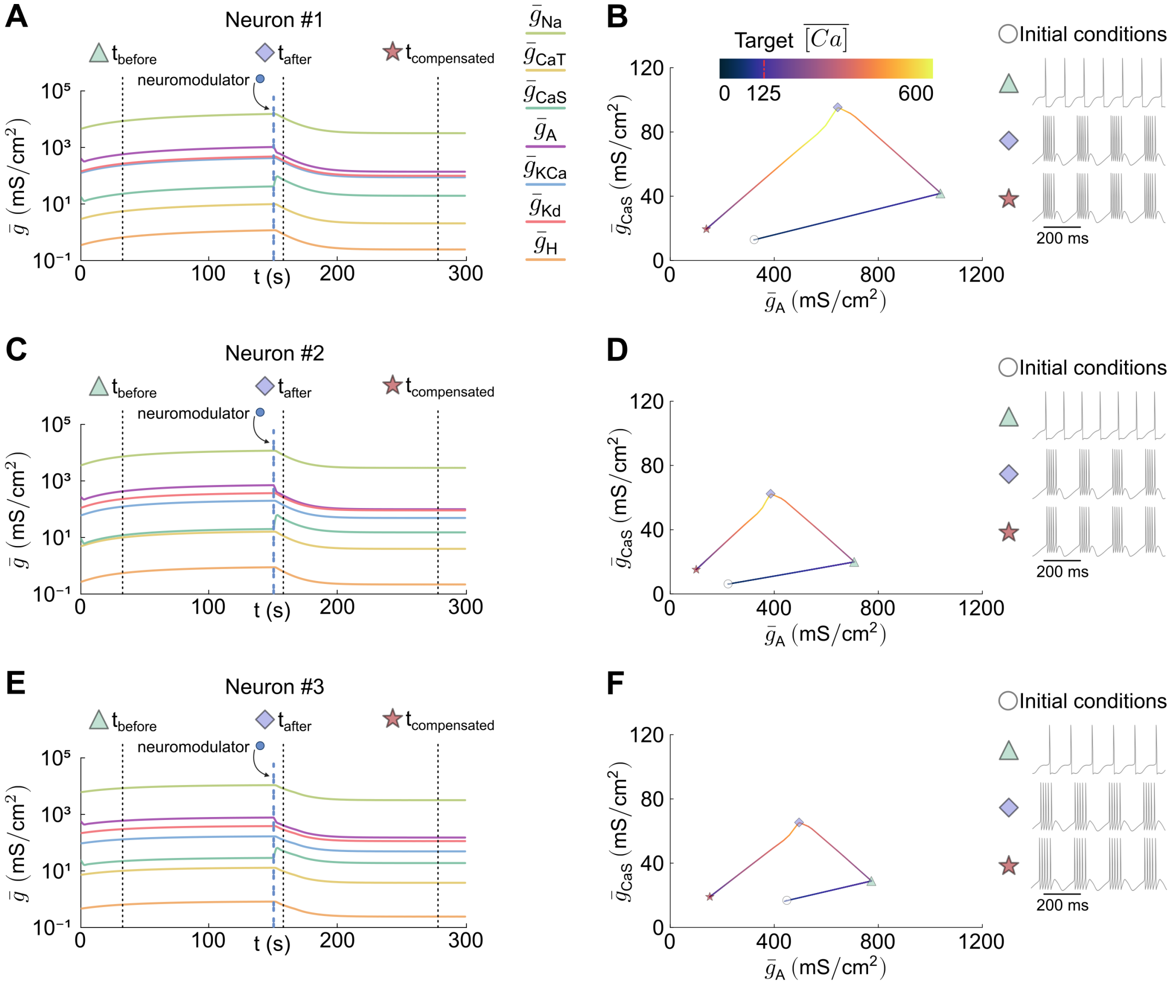

Fig S2. Single-neuron conductance trajectories under controlled neuromodulation with calcium homeostasis. Same three neurons as in Fig S1. **A, C, E.** Time evolution of all conductances on a logarithmic scale for each neuron. **B, D, F.** Corresponding trajectories in the modulated conductance plane ($\bar{g}_{\mathrm{CaS}}$, $\bar{g}_{\mathrm{A}}$), with color encoding the intracellular calcium concentration along the trajectory. Each row corresponds to one neuron. All three neurons converge to stable bursting with calcium levels near the homeostatic target.

model, the combination of controlled neuromodulation and calcium homeostasis reliably transitions the degenerate population of $N = 200$ DA neuron models from tonic spiking (pacemaking) to bursting while maintaining intracellular calcium at its target level.

I Neuromodulation of H-type channels

As discussed in the main text, H-type channels are well-established targets of neuromodulation through cAMP-dependent pathways [S10]. To confirm that our results are not specific to the particular subset of modulated

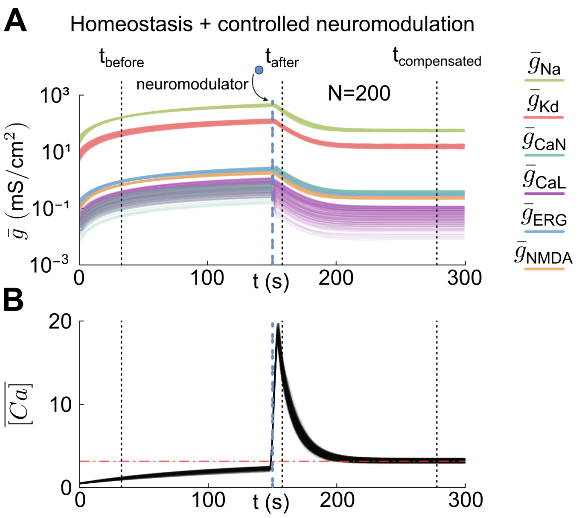

Fig S3. Controlled neuromodulation and calcium homeostasis naturally lead to robust and modulable neuronal function in the DA model. Same layout as Fig 2D–F of the main text, but for a degenerate population of $N = 200$ DA neuron models with controlled neuromodulation acting on $\bar{g}_{\mathrm{CaN}}$ and $\bar{g}_{\mathrm{CaL}}$. **A.** Time evolution of all conductances on a logarithmic scale. **B.** Corresponding mean intracellular calcium concentration over time; the target value is indicated by the red dash-dotted line. **C.** Three representative voltage traces at t_{before}, t_{after}, and $t_{\mathrm{compensated}}$ (top), and population activity distributions at these time points (bottom).

channels, we repeated the experiments of Fig 2 with controlled neuromodulation acting on $\bar{g}_{\mathrm{CaS}}$ and $\bar{g}_{\mathrm{H}}$ instead of $\bar{g}_{\mathrm{CaS}}$ and $\bar{g}_{\mathrm{A}}$.

The results are shown in Fig S4, which follows the same layout as Fig 2 of the main text. Panels A–C correspond to sharp neuromodulation combined with calcium homeostasis, while panels D–F correspond to controlled neuromodulation. The degenerate population of $N = 200$ STG neuron models is identical to that used in the main text.

Under sharp neuromodulation (Fig S4A–C), the same pattern of unreliable outcomes is observed: despite successful initial modulation from tonic spiking to bursting, calcium-homeostatic compensation produces highly variable results across the population, with many neurons losing bursting or entering irregular activity regimes. Under controlled neuromodulation (Fig S4D–F), the entire population reliably transitions to bursting and maintains this activity throughout calcium-homeostatic compensation, with intracellular calcium levels converging to the target value. These observations are qualitatively identical to those obtained when modulating $\bar{g}_{\mathrm{CaS}}$ and $\bar{g}_{\mathrm{A}}$ (Fig 2 of the main text), confirming that the constructive interaction between controlled neuromodulation and calcium homeostasis emerges from the activity-dependent nature of the control action rather than from the specific choice of modulated channels.

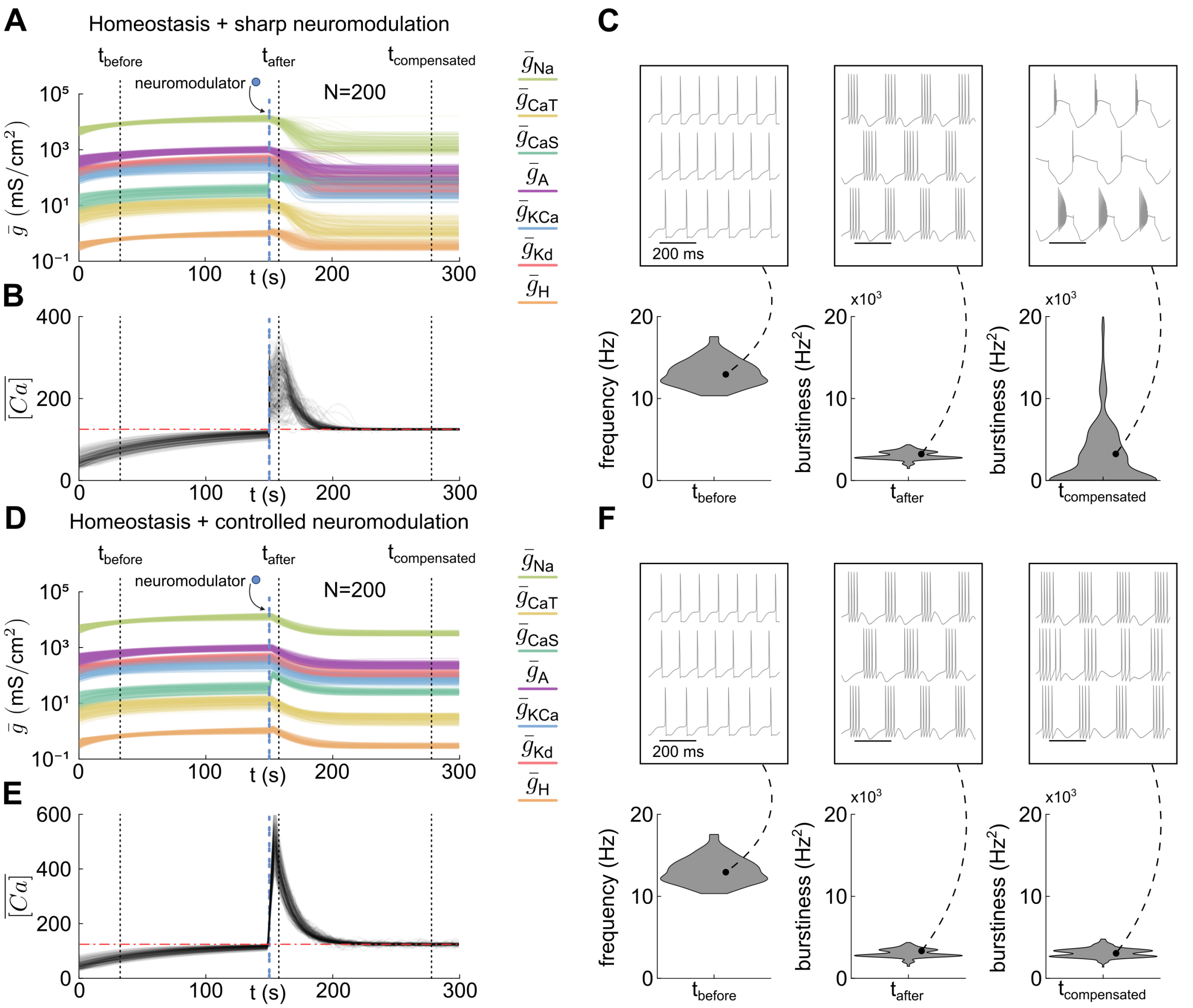

Fig S4. Neuromodulation of H-type channels yields the same qualitative results as modulation of A-type potassium channels. Same layout and population as Fig 2 of the main text, but with controlled neuromodulation acting on $\bar{g}_{\mathrm{CaS}}$ and $\bar{g}_{\mathrm{H}}$ instead of $\bar{g}_{\mathrm{CaS}}$ and $\bar{g}_{\mathrm{A}}$. **A.** Time evolution of all conductances on a logarithmic scale during homeostasis with sharp neuromodulation for a degenerate population of $N = 200$ neuron models. **B.** Corresponding mean intracellular calcium concentration over time; the target value is indicated by the red dash-dotted line. **C.** Three representative voltage traces at t_{before}, t_{after}, and $t_{\mathrm{compensated}}$ (top), and population activity distributions at these time points (bottom). **D–F.** Same as panels A–C, but with controlled neuromodulation.

J Neuromodulator washout dynamics

To complement the sustained neuromodulation results presented in the main text, we examine the response of the system when the neuromodulatory input is withdrawn (washout). Two scenarios are considered: washout shortly after neuromodulation is applied (at t_{after}, before calcium-homeostatic compensation has converged) and washout after the system has reached equilibrium (at $t_{\mathrm{compensated}}$). In both cases, the simulation is extended to 450 s to allow full compensation following washout, and we define t_{end} as the time point at which this post-washout compensation has converged.

All washout simulations use the same degenerate population of $N = 200$ STG neuron models and the same controller parameters as in Figs 2 and 3 of the main text. Neuromodulation is applied at $t = 150\,\mathrm{s}$ as in the main figures. For washout at t_{after}, the neuromodulatory input is removed at $t = 155\,\mathrm{s}$, shortly after the modulated conductances have reached their new values but before calcium-homeostatic compensation has had time to act. For washout at $t_{\mathrm{compensated}}$, the neuromodulatory input is removed at $t = 300\,\mathrm{s}$, after the system has reached equilibrium. In both cases, the simulation continues until $t = 450\,\mathrm{s}$ to allow full post-washout compensation. For sharp neuromodulation, washout resets $\bar{g}_{\mathrm{CaS}}$ and $\bar{g}_{\mathrm{A}}$ to the values they held immediately before neuromodulation was applied. For controlled neuromodulation, washout switches the DIC targets back to tonic spiking values, $(g_{\mathrm{s}}, g_{\mathrm{u}}) = (5, 4)$.

Sharp neuromodulation

Fig S5 shows the washout dynamics under sharp neuromodulation combined with calcium homeostasis, for the same degenerate population of $N = 200$ STG neuron models as in Fig 2 of the main text.

When washout occurs at t_{after} (Fig S5A–C), $\bar{g}_{\mathrm{CaS}}$ and $\bar{g}_{\mathrm{A}}$ are instantaneously reset to their pre-neuromodulation values, and the calcium-homeostatic controller continues to regulate intracellular calcium levels (panel B). The population reliably recovers tonic spiking at t_{end} (panel C). This recovery is expected, because calcium-homeostatic compensation had not yet had time to alter conductance correlations at t_{after}, the system essentially reverts to its original state.

When washout occurs at $t_{\mathrm{compensated}}$ (Fig S5D–F), tonic spiking is also recovered across the population (panel F). This outcome may appear surprising given the unreliable behavior observed during sustained sharp neuromodulation

(Fig 2A–C of the main text). However, as discussed in the main text and illustrated in Fig 3C, calcium-homeostatic compensation under sharp neuromodulation continuously drives conductances along the native tonic spiking scaling direction. Consequently, tonic spiking is the only remaining stable activity pattern, and upon washout the system has no other attractor to converge to. Importantly, however, the recovered state does not exactly match the pre-neuromodulation condition: because calcium homeostasis has been compensating for the sharp neuromodulatory perturbation throughout, the resulting conductance profiles are subtly altered compared to their original values. This manifests as a shifted and compressed distribution of firing frequencies across the population at t_{end} relative to t_{before} (panel F). Although the qualitative behavior (tonic spiking) is preserved, this quantitative drift implies that repeated cycles of sharp neuromodulation and washout could progressively erode the fidelity of neuronal function, as each cycle introduces small but cumulative changes in the conductance landscape.

Controlled neuromodulation

Fig S6 shows the washout dynamics under controlled neuromodulation combined with calcium homeostasis, for the same population.

When washout occurs at t_{after} (Fig S6A–C), the DIC targets are switched back to tonic spiking values, and the neuromodulation controller actively drives the modulated conductances toward the spiking regime. The calcium-homeostatic controller simultaneously restores intracellular calcium levels (panel B), and the population recovers tonic spiking (panel C).

When washout occurs at $t_{\text{compensated}}$ (Fig S6D–F), tonic spiking is again reliably recovered across the population (panel F). In this case, the system had been stably maintained in a bursting state by the combined action of both controllers. Upon washout, the DIC targets revert to spiking values, and the neuromodulation controller cooperates with the calcium-homeostatic controller to steer the system back to tonic firing.

The contrast between the two neuromodulation approaches is instructive. Under sharp neuromodulation, spiking is recovered because homeostatic compensation has already been pulling conductances toward the tonic spiking attractor throughout the simulation; no other stable state remains. However, when washout occurs at $t_{\text{compensated}}$, the recovered conductance distribution does not exactly match the original one, resulting in a subtly different firing frequency profile across the population. This residual drift, accumulated during the period of homeostatic compensation against the sharp perturbation, suggests that repeated neuromodulation-washout cycles could

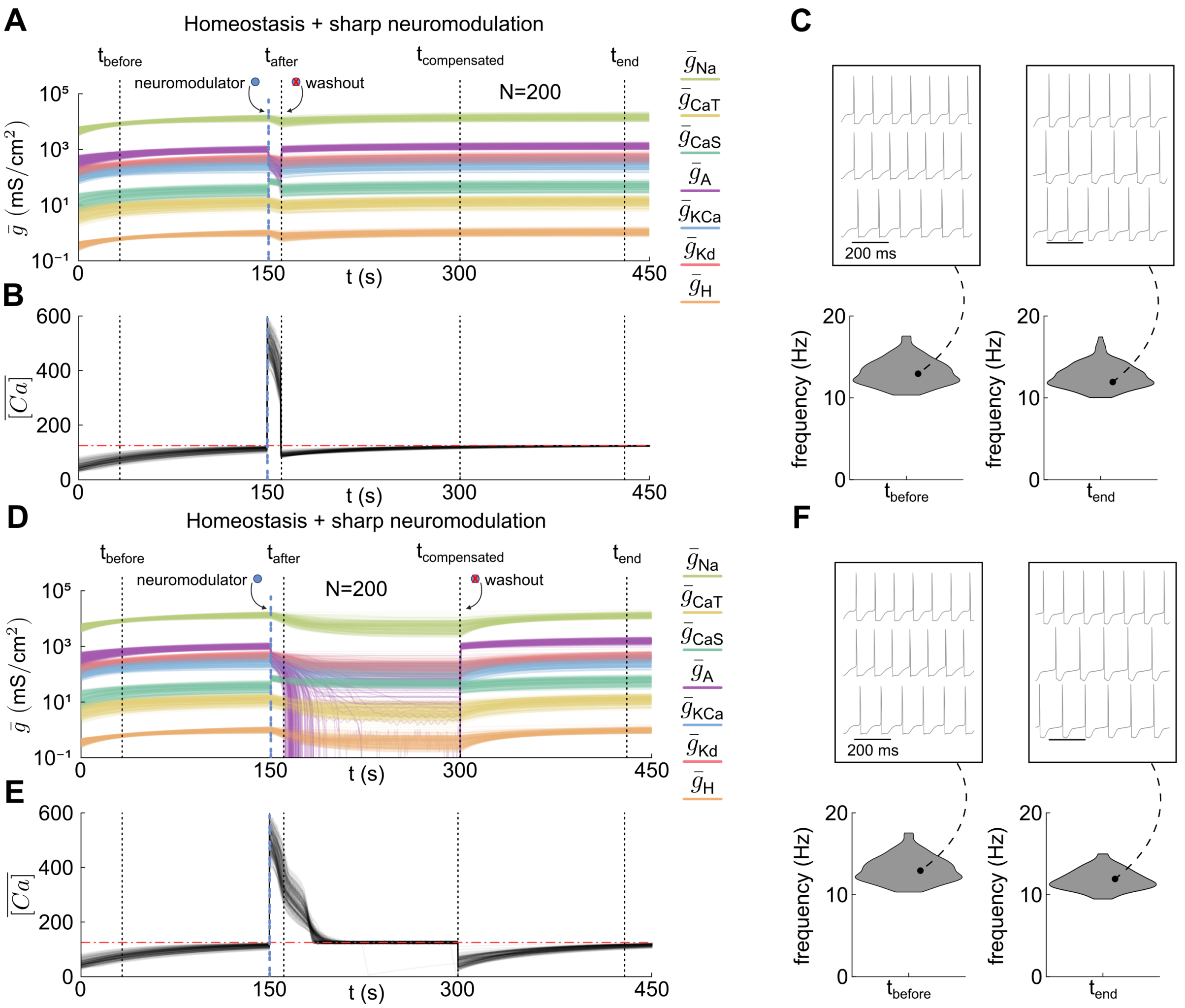

Fig S5. Washout dynamics under sharp neuromodulation with calcium homeostasis. Same population as Fig 2A–C of the main text. Washout is implemented by resetting $\bar{g}_{\mathrm{CaS}}$ and $\bar{g}_{\mathrm{A}}$ to their pre-neuromodulation values. **A–C.** Washout at t_{after}. **A.** Time evolution of all conductances on a logarithmic scale; the neuromodulatory input is removed at t_{after}, and t_{end} marks the end of post-washout compensation. **B.** Corresponding mean intracellular calcium concentration over time; the target value is indicated by the red dash-dotted line. **C.** Population firing frequency distributions at t_{before} and t_{end}. **D–F.** Same as panels A–C, but with washout at $t_{\mathrm{compensated}}$.

progressively degrade population-level functional homogeneity. Under controlled neuromodulation, spiking is recovered because the neuromodulation controller is actively redirected toward spiking DIC targets, and both controllers cooperate to return the system to its native operating point. Because controlled neuromodulation coordinates with calcium homeostasis rather than competing with it, the pre-neuromodulation conductance distribution is faithfully recovered upon washout, even at $t_{\mathrm{compensated}}$.

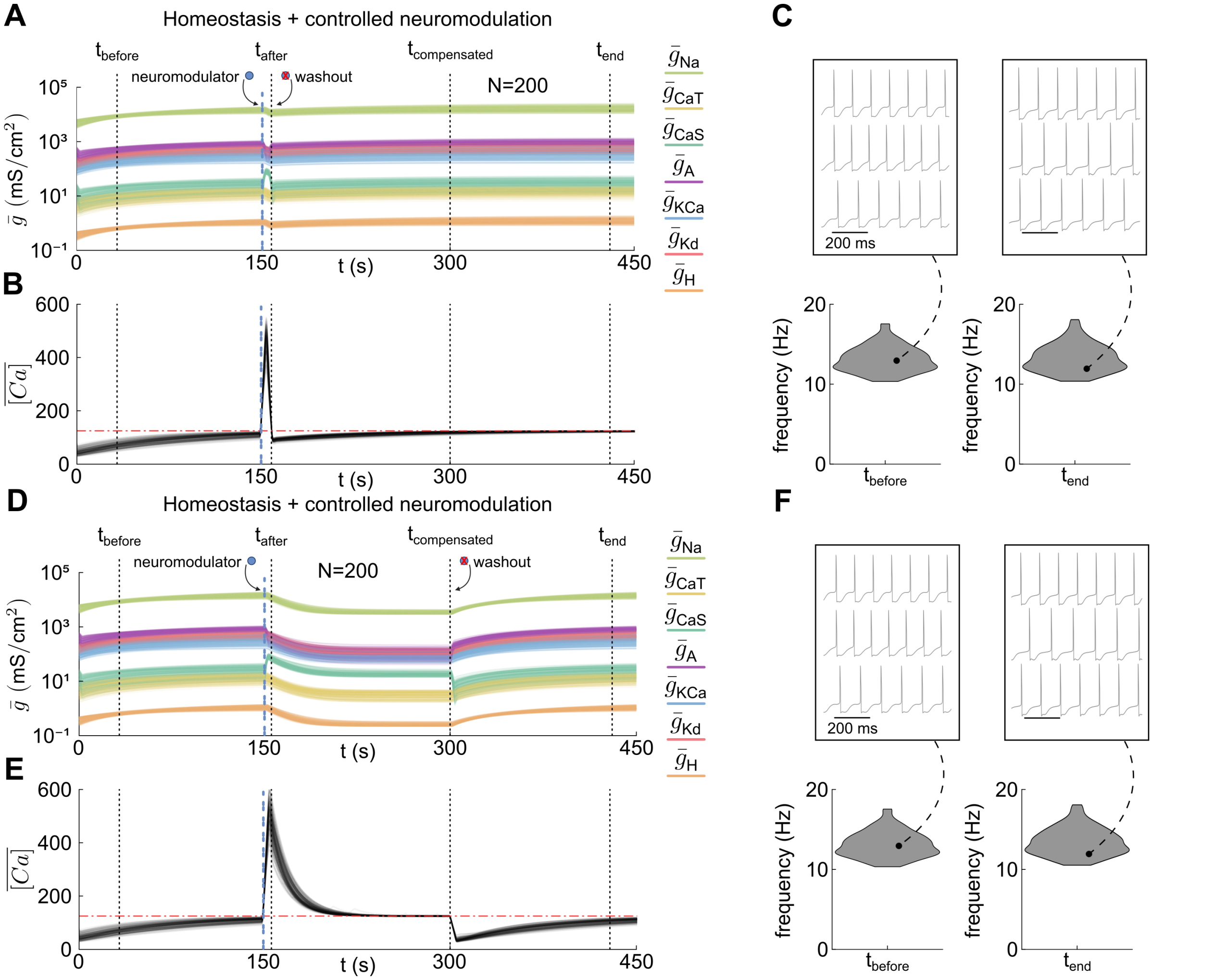

Fig S6. Washout dynamics under controlled neuromodulation with calcium homeostasis. Same population as Fig 2D–F of the main text. Washout is implemented by switching the DIC targets back to tonic spiking values. **A–C.** Washout at t_{after}. **A.** Time evolution of all conductances on a logarithmic scale; the neuromodulatory input is removed at t_{after}, and t_{end} marks the end of post-washout compensation. **B.** Corresponding mean intracellular calcium concentration over time; the target value is indicated by the red dash-dotted line. **C.** Population firing frequency distributions at t_{before} and t_{end}. **D–F.** Same as panels A–C, but with washout at $t_{\text{compensated}}$.

References

S1. Liu Z, Golowasch J, Marder E, Abbott L. A model neuron with activity-dependent conductances regulated by multiple calcium sensors. J Neurosci. 1998;18(7):2309-20. doi:10.1523/JNEUROSCI.18-07-02309.1998.

S2. Bezanson J, Edelman A, Karpinski S, Shah VB. Julia: A fresh approach to numerical computing. SIAM Rev. 2017;59(1):65-98. doi:10.1137/141000671.

S3. Qian K, Yu N, Tucker KR, Levitan ES, Canavier CC. Mathematical analysis of depolarization block mediated by slow inactivation of fast sodium channels in midbrain dopamine neurons. J Neurophysiol. 2014;112(11):2779-90. doi:10.1152/jn.00578.2014.

S4. Fyon A, Sacré P, Franci A, Drion G. Reliable neuromodulation from adaptive control of ion channel expression. IFAC-PapersOnLine. 2023;56(2):458-63. doi:10.1016/j.ifacol.2023.10.1610.

S5. Drion G, Franci A, Dethier J, Sepulchre R. Dynamic input conductances shape neuronal spiking. eNeuro. 2015;2(1). doi:10.1523/ENEURO.0031-14.2015.

S6. Fyon A, Franci A, Sacré P, Drion G. Dimensionality reduction of neuronal degeneracy reveals two interfering physiological mechanisms. PNAS Nexus. 2024;3(10):pgae415. doi:10.1093/pnasnexus/pgae415.

S7. Golub GH, Van Loan CF. Matrix Computations. 4th ed. Baltimore, MD: Johns Hopkins University Press; 2013. doi:10.56021/9781421407944.

S8. O'Leary T, Williams AH, Franci A, Marder E. Cell types, network homeostasis, and pathological compensation from a biologically plausible ion channel expression model. Neuron. 2014;82(4):809-21. doi:10.1016/j.neuron.2014.04.002.

S9. Gutierrez GJ, O'Leary T, Marder E. Multiple mechanisms switch an electrically coupled, synaptically inhibited neuron between competing rhythmic oscillators. Neuron. 2013;77(5):845-58. doi:10.1016/j.neuron.2013.01.016.

S10. Amendola J, Woodhouse A, Martin-Eauclaire MF, Goaillard JM. Ca2+/cAMP-sensitive covariation of IA and IH voltage dependences tunes rebound firing in dopaminergic neurons. J Neurosci. 2012;32(6):2166-81. doi:10.1523/jneurosci.5297-11.2012.
