# Supplementary material for: Activity-dependent neuromodulation and calcium homeostasis cooperate to produce robust and modulable neuronal function
Source: PLoS Comput Biol. 2026 Apr 15;22(4):e1014177. doi: 10.1371/journal.pcbi.1014177 (PMC13102308; doi:10.1371/journal.pcbi.1014177)
Supplement: Attachment — Submitted filename: Response to Reviewers.pdf [file pcbi.1014177.s002.pdf]

Response to reviewer comments for submission PCOMPBIOL-D-25-02581

Activity-dependent neuromodulation and calcium homeostasis cooperate to produce robust and modulable neuronal function

---

Dear Editor,
Dear Reviewers,

Please find enclosed the revision of our article "*Activity-dependent neuromodulation and calcium homeostasis cooperate to produce robust and modulable neuronal function*".

Below, we provide detailed responses to all the reviewers' comments. We reproduced each of the *reviewers' comments in blue italics* and give our responses in black. Major modifications in the revised manuscript are marked in dark orange and are referred to in the responses below as appropriate.

The most significant changes to the manuscript are the following.

- **Restructured Results section.** Following the Editor's comments on clarity, we reorganized the first two Results subsections. The first subsection now describes the two regulatory mechanisms (calcium homeostasis, and neuromodulation in both its sharp and controlled forms) before any simulation results, with the schematics figure presented first (now Fig 1). The second subsection presents a side-by-side comparison of sharp versus controlled neuromodulation interacting with calcium homeostasis, combining the former Figs 1 and 3 into a single Fig 2 (panels A–C for sharp, D–F for controlled neuromodulation). All cross-references in the manuscript have been updated to reflect this new structure.
- **Expanded biological grounding of controlled neuromodulation.** Following comments from Reviewers 2 and 3, we have added concrete proposals for biological signaling pathways that could plausibly implement controlled neuromodulation, and expanded the discussion to better connect our findings to broader biological contexts, as also requested by the Editor.
- **Inclusion of Ih currents in the neuromodulatory framework.** In response to Reviewer 3's major comment, we have conducted additional experiments incorporating Ih channels as neuromodulatory targets, given their well-established sensitivity to cAMP levels and their role in pacemaker currents. These supplementary results confirm the generality of the framework.
- **Analysis of neuromodulator washout dynamics.** Following Reviewer 3's second major comment and related remarks by Reviewer 1, we have addressed the tempo-

ral dynamics of neuromodulatory perturbations, including what happens when the neuromodulator is withdrawn after the system reaches a new equilibrium.

- **Clarified the inner workings of the controlled neuromodulation mechanism.** Following Reviewer 2's first major comment, we have added more intuitive explanations and concrete examples in the main text to describe how controlled neuromodulation operates, including clearer decomposition of the feedforward and feedback contributions.

- **Discussion of the limits of homeostatic compensation.** In response to Reviewer 2's third major comment, we have clarified the intended message of Figure 5 and discussed what constrains compensation in real neurons, connecting our results to the existence of neurological and neurodegenerative conditions.

- **Substantially improved figures.** Following detailed comments from all three reviewers, we have revised all main figures with improved axis labels, scales, and annotations. We have also added single-neuron example traces (Reviewer 1), improved schematics (Reviewers 1 and 3), and clarified the raster-like plots in Figs 3 and 5.

- **Revised terminology and naming conventions.** Following Reviewer 3's suggestions, we now consistently use "calcium-homeostatic controller" throughout the manuscript.

- **Reorganized the DA neuron section.** Following Reviewer 3's suggestion, we have moved the DA neuron case study from the Results to the Discussion to improve the flow of the manuscript.

We hope that these revisions adequately address the concerns raised by the Editor and the Reviewers.

We are grateful to the reviewers for their thorough feedback that allowed us to improve the quality of the manuscript. We hope that our revisions meet your and the reviewers' satisfaction, and we look forward to hearing from you at your earliest convenience.

Kind regards,

The authors

Response to Additional Editor Comments

"The manuscript has merit, but requires significant revision to address several concerns. The biological relevance remains somewhat limited, and the authors would benefit from expanding the discussion to better connect their findings to broader biological contexts. Additionally, the analysis lacks clarity and needs more detailed explanation to guide the reader."

We thank the Editor for their assessment and guidance. We have addressed both concerns in the revised manuscript.

Regarding biological relevance, we have substantially expanded the biological grounding of controlled neuromodulation. We now provide concrete examples of signaling pathways that could plausibly implement the proposed mechanism, including the convergence of multiple neuromodulators onto the modulator-activated inward current I_{MI} in the STG [42], and experimental evidence for activity-dependent neuromodulation [2, 34–38]. We have also added a new paragraph in the Discussion connecting our results to neurological and neurodegenerative conditions (channelopathies, Parkinson's disease), and demonstrated the generality of the framework on a midbrain dopaminergic neuron model. Furthermore, in response to Reviewer 3, we conducted additional experiments with Ih channels as neuromodulatory targets, confirming that the results are not specific to a particular subset of modulated channels.

Regarding clarity, we have reorganized the Results section so that the first subsection now describes the two regulatory mechanisms (calcium homeostasis, sharp and controlled neuromodulation) with the schematics figure (Fig 1) before any simulation results, improving the logical flow. The second subsection then presents a side-by-side comparison of both neuromodulation types. We have also added detailed explanations of how the controlled neuromodulation mechanism operates, including a decomposition of its feedforward and feedback components (addressing Reviewer 2's first major concern). We have substantially revised all figures with improved axis labels, single-neuron example traces, population activity distributions, and clearer schematics. Finally, we have adopted consistent terminology throughout the manuscript ("calcium-homeostatic controller", "sharp" versus "controlled" neuromodulation) and added a washout analysis to address questions about the temporal dynamics of neuromodulation.

Changes in manuscript: The specific changes addressing each of these points are detailed in the individual reviewer responses below.

Response to Reviewer 1

We thank Reviewer 1 for their thorough and constructive comments. Their feedback was highly relevant and greatly contributed to elevating the clarity, rigor, and scope of our manuscript. We believe that addressing these comments has substantially strengthened the paper.

R1.1 *"The figures shown in Fig. 1, 2, 3 should have specific labels on the scales beyond the maximum values, especially we can detect how much changes are there in the x-axis and y-axis, and how much difference between different lines. Since it seems that there are so much difference between the different channel conductance, and even within the same channel (the same-colored curve), it seems in the logarithm scale there are also so much difference. So, in biological perspective, are these differences physiologically meaningful or making sense?"*

We thank the Reviewer for raising this important point. We have revised all figures to include clearer axis labels and tick marks, allowing readers to assess the magnitude of conductance changes and differences between channels.

Regarding the biological meaningfulness of the observed conductance differences, we have added a paragraph in the Results section to address this directly. Different ion channel types are known to have effective conductances spanning several orders of magnitude [29, 43], which is why logarithmic scale is appropriate for visualizing them in a single graph. Moreover, the effective conductance of a given ion channel type can vary severalfold within a single neuron type [44]. The variability in our dataset is inspired by these biological measurements and slightly exceeds the observed range to test the limits of our method. These large differences are therefore not artifacts of the model but reflect the known heterogeneity of ion channel expression in biological neurons.

Changes in manuscript:

- *(All figures)* Added tick marks on all axes throughout Figs 1–6. Corrected the unit of burstiness from Hz to Hz^2 in all relevant figures.
- *(Results, second subsection)* Added: "Note that these differences in channel conductances are consistent with biological measurements: different channel types have effective conductances spanning several orders of magnitude [29, 43]. Moreover, the effective conductance of a given ion channel type can vary severalfold within a single neuron type [44]. The variability in the dataset from [33] is inspired by these biological measurements and slightly exceeds the observed range to test the robustness of our analysis."

R1.2 *"In Fig. 1 and Fig. 2, it is better to show one specific example among the 200 models, how each conductance in 1 model is changing with application of neuromodulation (both sharp and controlled modulation), what's the transient state about the spiking activity instead of the dashed dotted lines, and how the homeostasis changes each conductance, instead of showing the calcium dynamics only."*

We agree that showing individual neuron trajectories greatly improves interpretability. We have made two changes in response. First, the revised Fig 2 now includes panels C and F that highlight three representative neurons from the population, showing their voltage traces at three key time points: before neuromodulation (t_{before}), immediately after (t_{after}), and after calcium-homeostatic compensation ($t_{\text{compensated}}$). Panel C shows these traces for sharp neuromodulation and panel F for controlled neuromodulation, allowing direct comparison. This enables the reader to observe the transient spiking activity and how individual neurons respond differently to each type of neuromodulation and homeostatic compensation. Second, we have added de-

tailed single-neuron time evolution curves (showing all individual conductances over time) to Supporting Information, S1 Appendix, for both the sharp and controlled neuromodulation cases. These supplementary figures complement the population-level view in the main figures by providing the full conductance dynamics for selected neurons.

Note that as part of the revision, we have restructured the Results section: the former Figs 1 and 3 (sharp and controlled neuromodulation results, respectively) have been combined into a single Fig 2, with panels A–C for sharp neuromodulation and panels D–F for controlled neuromodulation. The schematics figure (formerly Fig 2) is now Fig 1 and precedes the simulation results.

Changes in manuscript:

- *(Fig 2, panels A–C; formerly Fig 1)* Added panel C with three representative single-neuron voltage traces at t_{before}, t_{after}, and $t_{\text{compensated}}$, along with population activity distributions at these time points.
- *(Fig 2, panels D–F; formerly Fig 3)* Added an analogous panel F for controlled neuromodulation.
- *(Results, second subsection)* Added: "Specific time evolution curves for single neurons within the population can be found in Supporting Information, S1 Appendix."
- *(S1 Appendix)* Added supplementary figures showing individual conductance trajectories for selected neurons under both sharp and controlled neuromodulation.

R1.3 *"The difference between sharp modulation and controlled modulation is only the time period that been applied to the neuron. Then how long the controlled modulation is kind of important. What will the dynamic of the system look like if the controlled modulation withdrawn after the balance between it and homeostasis controlled have been reached? The different driving force might be mediated by the calcium homeostasis by changing other calcium dependent channel conductance, it will be necessary to clarify the tonic spiking and bursting behavior by detailed dynamic analysis with the different combinations of stimuli. Minor: the burstiness in Hz shown in the Fig.c is also logarithm? The detailed scale should be marked on the y-axis; how do you define the frequency? The inter-burst-interval? Or the spike frequency within one burst?"*

We thank the Reviewer for this insightful question, which prompted us to clarify several important points. First, we want to clarify that the difference between sharp and controlled neuromodulation is not merely the duration of application but rather the nature of the mechanism itself. Sharp neuromodulation applies an activity-

independent change to a subset of conductances. Controlled neuromodulation, by contrast, is activity-dependent: it continuously senses the neuronal output and adjusts modulated conductances through a feedback loop to reach a desired firing pattern. Only controlled neuromodulation adapts its action based on the current state of the neuron.

Regarding neuromodulator washout, we have added a new analysis in the revised manuscript and in Supporting Information, S1 Appendix. Briefly, washout recovers tonic spiking across the population under both sharp and controlled neuromodulation, at both t_{after} and $t_{\text{compensated}}$, though for fundamentally different reasons and with different levels of fidelity. Under sharp neuromodulation, calcium-homeostatic compensation continuously drives conductances along the native tonic spiking scaling direction, so that tonic spiking is the only remaining stable activity pattern; recovery upon washout is therefore expected, as the system has no other stable attractor. However, at $t_{\text{compensated}}$, the recovered conductance distribution does not exactly match the pre-neuromodulation state, resulting in a subtly shifted firing frequency profile across the population. This quantitative drift suggests that repeated cycles of sharp neuromodulation and washout could progressively degrade functional fidelity. In contrast, under controlled neuromodulation, the system can maintain any target activity through activity-dependent feedback; upon washout, the neuromodulatory reference is removed, and the system returns to tonic spiking because the calcium-homeostatic controller regains sole authority over conductance tuning. Because both controllers operate in coordination rather than in competition, the pre-neuromodulation state is faithfully recovered. We note that the primary focus of this study is on sustained neuromodulation, as many neuromodulatory states in biological systems are maintained over extended periods by tonic release from projection neurons [46, 47].

Regarding the burstiness metric, we have clarified its definition in the revised Fig 2 caption. Burstiness is defined following [45] as the product of the intraburst frequency (reciprocal of the interspike interval within a burst), the interburst frequency (reciprocal of the interburst interval), and the number of spikes per burst. Before neuromodulation, when the population exhibits tonic firing, we use the firing frequency (reciprocal of the interspike interval) as the activity metric instead. A burstiness of zero indicates that the neuron is not producing multi-spike bursts. The burstiness axis in the violin plots is expressed in Hz^2, consistent with the definition above (a product of two frequencies and a dimensionless spike count), and is displayed on a linear scale.

Changes in manuscript:

- *(Results, second subsection)* Added a paragraph on washout dynamics: "The dif-

ferences between sharp and controlled neuromodulation can also be highlighted through neuromodulator washout. . . ”

- *(Fig 2 caption)* Added explicit definition of burstiness and clarified the distinction between the firing frequency and burstiness metrics used before and after neuromodulation.
- *(S1 Appendix)* Added washout figures for both sharp and controlled neuromodulation at t_{after} and $t_{\text{compensated}}$.

R1.4 *“In Fig. 4, how the black curve happened? The title in A: Homeostasis controller “alone” with sharp neuromodulation, might be “along”?”*

We thank the Reviewer for this question. In Fig 4, the black curve (arrow) represents the trajectory of the neuromodulatory action in the modulated conductance plane, that is, the change in $\bar{g}_{\text{CaS}}$ and $\bar{g}_{\text{A}}$ applied by neuromodulation. In the sharp neuromodulation case (Fig 4A), this corresponds to an instantaneous jump in these conductances. In the controlled neuromodulation case (Fig 4B), it corresponds to the gradual adjustment driven by the neuromodulation controller. The red arrows then represent the subsequent calcium-homeostatic compensation. We have clarified the meaning of all arrows in the revised figure caption by adding an explicit legend.

Regarding "alone" versus "along": the original manuscript indeed used "alone" intentionally, to convey that calcium homeostasis acts without the neuromodulation controller in the sharp neuromodulation case. However, we agree that this phrasing was confusing. In the revised manuscript, Fig 3 has been restructured with separate panels C and D for sharp and controlled neuromodulation, respectively, and the schematic has been moved to its own Fig 4 with clearer labeling, avoiding this ambiguity.

Changes in manuscript:

- *(Fig 4)* Restructured the figure with clearer panel labeling, removing the ambiguous “alone” phrasing, and added an explicit arrow legend in the caption: “Black arrow: trajectory of sharp (A) or controlled (B) neuromodulation. Solid red arrow: trajectory of calcium-homeostatic compensation. Dashed red arrow: direction of the calcium-homeostatic compensation in tonic firing (A).”
- *(Fig 3)* Split panel C into two separate panels (C and D) for sharp and controlled neuromodulation, respectively.

R1.5 *“In Fig. 5 the y-axis should be labeled with different scale such that readers will know how much frequency they are distributed in. Can the author give specific explanations on why the different channel type blockage leads to different physiological and compensational results?”*

We have revised Fig 5 to include properly labeled y-axes on the activity distribution plots (violin plots), so that the reader can directly assess the range and distribution of burstiness across the population at each time point.

Regarding the different outcomes of channel blockades, we have substantially expanded the explanations in the revised manuscript. In brief, the key factor determining whether a blockade is compensable is the degree of degeneracy of the blocked channel, that is, whether other channels in the model can substitute for its functional role:

- H-type channels (Fig 5A): their blockade does not disrupt bursting, because their contribution to the bursting mechanism can be readily compensated by other slow and ultraslow channels in the model.
- T-type calcium and KCa channels (Fig 5B-C): despite operating at different timescales and membrane potentials, and thus producing different acute effects upon blockade, recovery is possible because their functions overlap with those of other channels, that is, they exhibit degeneracy [31]. However, the function of KCa channels is less degenerate, as reflected in the reduced population burstiness even after compensation.
- Sodium channels (Fig 5D): their blockade is irrecoverable because they are essential for spike initiation on a fast timescale that no other channel in the model can replicate. This function is non-degenerate.

Changes in manuscript:

- *(Fig 5)* Revised violin plots with explicit y-axis labels and scales.
- *(Results, fourth subsection)* Expanded the explanation of differential blockade outcomes, including the paragraph: "Despite operating at different timescales and membrane potentials, and thus producing different acute physiological effects upon blockade, recovery is possible for these channels because their functions overlap with those of other channels in the model, *i.e.*, they exhibit degeneracy..."

R1.6 *"In Fig. 6, the Fig.A x-axis with multiplication of different numbers, what does that mean? Also, the neuromodulation seems only change the slow rhythm, since there is a red neuron connecting between fast rhythm and slow rhythm, how strong the neuromodulation will change the purple and blue neuron firing activates and which factors will affect the patterns that system could generate?"*

We thank the Reviewer for this question. In the original Fig 6A, the x-axis displayed the initial conductance values of each neuron in the network, with multiplier coefficients applied to all conductances to facilitate visualization in a non-logarithmic

graph. This representation follows the convention introduced in [43] and allows direct comparison of the different initial conductance values across all five neurons. In the revised manuscript, this information has been moved to a dedicated panel (Fig 6B) with an improved caption that explicitly explains the meaning of the multiplier coefficients.

Regarding the effect of neuromodulation on the fast rhythm neurons (N1 and N2, purple and blue), we have added a detailed mechanistic paragraph in the revised Results section. In our model, neuromodulation primarily targets the slow-rhythm neurons (N4 and N5) to activate the gastric mill rhythm, while the fast-rhythm neurons are not directly neuromodulated. The fast rhythm remains active throughout, with only minor phase adjustments resulting from the synaptic coupling to the slow-rhythm neurons via the central neuron (N3). The factors that determine which rhythmic patterns the network can generate include the intrinsic bursting properties of each neuron (duty cycle, burst duration, and frequency), the strength and sign of synaptic connections, and the neuromodulator concentration applied to the slow-rhythm neurons. A systematic exploration of the parameter space governing network pattern generation is beyond the scope of this study but represents an interesting direction for future work.

Changes in manuscript:

- *(Fig 6)* Moved the conductance bar chart to a dedicated panel B with an improved caption explaining the multiplier coefficients.
- *(Results, fifth subsection)* Added a mechanistic paragraph explaining the network-level dynamics, including: "The fast rhythm (N1 and N2, purple and blue) remains active throughout, with only minor phase adjustments resulting from the electrical coupling to the slow-rhythm neurons. This is consistent with the experimental observation that pyloric rhythm modulation during gastric mill activation mainly involves changes in phase relationships..."

Response to Reviewer 2

We thank Reviewer 2 for their careful reading of the manuscript and for their thoughtful and constructive feedback. Their comments helped us improve the clarity, biological grounding, and explanatory depth of our work. We believe that addressing these comments has substantially strengthened the paper.

Major concerns

R2.1 *"In the current form, the manuscript describes the behavior of models under controlled neuromodulation more than delineating "inner workings" by which controlled neuro-*

modulation is implemented. I understand that the method has been published previously (ref. 30) and that additional details are provided in the Methods section. Nevertheless, I recommend adding more explanation in the main text of how controlled neuromodulation operates, ideally through concrete examples and intuitive descriptions that would help a broad readership. For example, though I do find Figure 4 helpful, it also primarily focuses on model behavior. It may be more informative to show how distinct components of the model (e.g., feedforward vs. feedback) contribute to achieving the model behavior."

We thank the Reviewer for this suggestion, which we agree was a significant gap in the original manuscript. We have added two new paragraphs in the Results section that provide an intuitive, step-by-step description of how the controlled neuromodulation mechanism operates. As part of the restructuring, these paragraphs now appear in the first Results subsection, which describes the two regulatory mechanisms before any simulation results are shown.

The first paragraph explains the two-stage architecture of the controller. The feedforward step exploits Dynamic Input Conductances (DICs), a low-dimensional representation that links ion channel conductances to firing patterns through a linear mapping. Given a desired firing pattern and the current state of all unmodulated conductances, this mapping computes neuron-specific reference values for the modulated conductances. The feedback step then uses a Proportional-Integral (PI) controller to track these references by regulating ion channel expression. Because the feedforward step depends on the full conductance state of the neuron, the controller naturally adapts its output to each individual neuron within a degenerate population: different neurons receive different conductance adjustments, yet all converge to the same target activity.

The second paragraph clarifies that the controller is agnostic to the specific neuromodulator identity and signaling cascade involved, and explains why this abstraction is justified. In the STG, for instance, multiple neuromodulators acting through distinct G-protein-coupled receptors and second messenger pathways all converge onto the same modulator-activated inward current I_{MI} [42]. Our controller captures the functional principle common to these diverse pathways: an activity-dependent feedback loop that senses neuronal output and adjusts a targeted subset of conductances.

We believe that the schematic explanation of Fig 4 now also better serves the purpose of illustrating how the two components interact. The feedforward component determines where the neuron should go in conductance space (the target activity isocline), while the feedback component ensures it stays there despite homeostatic drift. The sawtooth trajectory in Fig 4B directly visualizes this interplay: calcium homeostasis pulls the neuron off the isocline, and the fast neuromodulatory feedback corrects the deviation.

Changes in manuscript:

- *(Results, first subsection)* Added two new paragraphs: the first beginning with "At its core, the controller exploits Dynamic Input Conductances (DICs)..." describing the feedforward/feedback architecture; the second beginning with "Importantly, the controller is agnostic to the specific neuromodulator identity..." explaining the biological justification for the abstraction.
- *(Fig 4)* Revised caption to clarify the roles of the black arrow (feedforward neuromodulatory action), solid red arrow (homeostatic drift), and sawtooth arrow (feedback correction by controlled neuromodulation).

R2.2 *"I am not yet fully convinced that controlled neuromodulation, as defined here, can be realized by known biological mechanisms. The manuscript would benefit from concrete proposals or examples of signaling pathways that could plausibly implement controlled neuromodulation."*

We thank the Reviewer for pressing us on this important point. We have expanded the manuscript in two places to provide concrete biological grounding for controlled neuromodulation.

First, in the Results section, we now discuss the convergence of diverse neuromodulatory signaling pathways onto shared functional targets as experimental support for our framework. A well-characterized example is the modulator-activated inward current I_{MI} in the STG, onto which multiple neuromodulators acting through distinct G-protein-coupled receptors and second messenger pathways (involving cAMP or calcium-dependent cascades) all converge [42]. This convergence suggests that the nervous system implements a form of functional abstraction similar to the one captured by our controller: different molecular pathways produce equivalent effects on neuronal excitability, consistent with an activity-dependent feedback principle rather than a pathway-specific open-loop action.

Second, the activity-dependence that is central to controlled neuromodulation is well documented experimentally. Neuromodulatory effects are known to depend on the ongoing activity state of the target neuron [34–37]. For instance, Kramer and Levitan [34] showed that the same neuromodulator can produce qualitatively different effects depending on the baseline activity of the neuron, which is precisely the property captured by our controller through the DIC-based feedforward step. The feedback step (PI controller) models the temporal integration inherent to intracellular signaling cascades, where second messenger concentrations gradually adjust in response to sustained receptor activation.

We emphasize that our controller is not intended to model any particular signaling cascade in molecular detail. Rather, it captures the shared functional logic,

namely activity-dependent feedback, that underlies diverse neuromodulatory pathways. The biological plausibility of the framework rests on this functional correspondence rather than on a one-to-one mapping to specific molecular components.

Changes in manuscript:

- *(Results, first subsection)* Added the paragraph beginning with "Importantly, the controller is agnostic to the specific neuromodulator identity. . . ", including the I_{MI} convergence example and additional references [36, 37, 42].
- *(Fig 1B)* Revised the schematic of controlled neuromodulation to emphasize the activity-dependent feedback loop and its biological counterparts in the G-protein-coupled receptor cascade. Added BioRender attribution.
- *(Discussion, second paragraph)* Expanded the biological grounding with additional references to activity-dependent neuromodulation.

R2.3 *"I find Figure 5 and the related discussion quite amazing, but they raise questions, especially in relation to Concern 2, about what limits such compensation in real neurons. If compensation can be pushed arbitrarily far as in the model, there should be no neurological and/or neurodegenerative conditions caused by ion-channel deficiencies or malfunctions as they can be compensated. In that regard, I am not entirely sure what the authors intend to deliver with Fig. 5."*

We thank the Reviewer for this thought-provoking comment, which helped us sharpen the message of this section considerably.

The intended message of Fig 5 is precisely that compensation is not always possible, and that the outcome depends on the degree of degeneracy of the blocked channel. The figure presents a gradient of outcomes: from fully compensable blockades (H-type and CaT channels, Fig 5A-B), through partially compensable ones (KCa channels, Fig 5C), to completely irrecoverable ones (Na channels, Fig 5D). This gradient directly illustrates the limits of the framework and, by extension, the biological limits of homeostatic compensation.

Several factors constrain compensation in both our model and in real neurons:

- **Non-degenerate channel functions.** When a blocked channel performs a unique role that no other channel can replicate (as with sodium channels for spike initiation), compensation is impossible regardless of the control input. This is a hard biological constraint.
- **Partial degeneracy.** When the function of a blocked channel partially overlaps with others but is not fully redundant (as with KCa channels), compensation may restore the qualitative behavior (bursting) but not the quantitative prop-

erties (burstiness). This is reflected in the reduced population burstiness after KCa blockade compensation.

- **Need for neuromodulator adjustment.** Even when compensation is theoretically possible, the neuron may not reach the compensated state without a change in neuromodulator concentration. In the KCa blockade case, the feedback gains had to be tuned, corresponding to a different neuromodulator cocktail. In a biological context, this would require the neuromodulatory system to adapt its output in response to the channel loss, a process that may or may not occur.

- **Absence of a convergence point.** As discussed in the revised Discussion, the success of the tandem of controllers depends on the existence of an intersection between the target activity isocline and the calcium homeostasis setpoint in conductance space. When no such intersection exists, neither controller can achieve its objective.

These constraints provide a direct link to neurological and neurodegenerative conditions. Channelopathies, for instance, involve mutations that impair or eliminate specific ion channel functions. Our framework predicts that the clinical severity of such conditions would depend on the degree of degeneracy of the affected channel: mutations affecting highly degenerate channels may be largely compensated and produce mild phenotypes, whereas mutations affecting non-degenerate channels would lead to severe dysfunction. Similarly, the progressive loss of neuromodulatory input in neurodegenerative diseases (such as dopamine depletion in Parkinson's disease) could be interpreted within our framework as a gradual loss of the controlled neuromodulation component, leaving the calcium-homeostatic controller to operate alone, potentially leading to the pathological compensations described in our sharp neuromodulation results (Fig 2A-C).

We have revised the Discussion to make these points explicit.

Changes in manuscript:

- *(Results, fourth subsection)* Expanded the discussion of KCa blockade compensation limits: "However, even when the blockade is theoretically compensable, the control input may need to be adjusted. In the case of the KCa channel blockade, the loss was compensated by tuning the neuronal feedback gains, which corresponds to applying a different neuromodulator concentration to partially recover the function. This highlights that, even when compensation is theoretically possible, the neuron may not reach the compensated state without adjusting the neuromodulator cocktail."

- *(Discussion)* Added: "Moreover, the mere existence of such an intersection point

does not guarantee that a neuron will reach it. For instance, to compensate for the loss of KCa channels (Fig 5C), a different neuromodulation target was required, corresponding to a different neuromodulator concentration. This means that, even when the intersection point exists, the neuron may require a specific neuromodulator concentration (or cocktail of neuromodulators) to partially or fully restore its function."

- *(Discussion, new paragraph before pharmacological implications)* Added a paragraph connecting the limits of compensation to clinical conditions: "These constraints on compensation have direct implications for understanding neurological and neurodegenerative conditions. Our framework predicts that the clinical severity of channelopathies [. . .] would depend on the degree of degeneracy of the affected channel [. . .]. Similarly, the progressive loss of neuromodulatory input characteristic of neurodegenerative diseases, such as dopamine depletion in Parkinson's disease, can be interpreted within our framework as a gradual loss of the controlled neuromodulation component [. . .]."
- *(Discussion, last paragraph)* Extended the pharmacological implications paragraph to include: "If neuronal degeneracy is insufficient (or if the amount of neuromodulator released is unchanged), such interventions may compromise the ability to sustain robust neuromodulation."

Minor comments

R2.m1 *"Page 5, lines 90–92 ("to facilitate . . . original implementation"). The meaning of this phrase is unclear to me."*

We apologize for the unclear phrasing. The sentence was intended to convey that, for visualization purposes, the time constant of the calcium-homeostatic controller was reduced so that both neuromodulation and homeostatic compensation could be observed within a single simulation trace. We have rewritten this parenthetical remark to make its meaning explicit.

Changes in manuscript: *(Results, second subsection)* Changed to a standalone sentence: "In all illustrative simulations (Fig 2), the time constant of the calcium-homeostatic controller was reduced so that the dynamics of both neuromodulation and calcium-homeostatic compensation could be observed within a single trace, without affecting the qualitative behavior of the system."

R2.m2 *"Page 6, line 124 ("cyclic amp"). Please spell out "cyclic AMP (adenosine monophosphate)" at first use."*

We thank the Reviewer for catching this. We have spelled out the full name at first use as suggested.

Changes in manuscript: *(Results, first subsection)* Changed: "using an intermediate signal such as *e.g.* cyclic amp" to: "using intermediate signals such as, *e.g.*, cyclic adenosine monophosphate (cAMP) or intracellular calcium".

R2.m3 *"Could the authors comment on how controlled neuromodulation can be extended to work in the context of dendritic conductances and compartmentalization?"*

We thank the Reviewer for this thought-provoking question. The controlled neuromodulation framework, as presented in this work, operates on single-compartment models where all conductances are co-localized. Extending it to multi-compartment models with dendritic conductances is conceptually straightforward but raises practical challenges that we now discuss in the revised manuscript.

Since the neuromodulation controller relies on Dynamic Input Conductances (DICs), which are computed from the linearization of the full model around a reference voltage, the extension to compartmental models would require computing DICs for each compartment while accounting for the electrotonic coupling between them. In principle, the DIC framework naturally extends to multi-compartment settings, as the sensitivity matrix $S(V)$ can be generalized to include the contributions of dendritic conductances and inter-compartmental coupling conductances. However, several challenges arise. First, the dimensionality of the problem increases substantially, as detailed multi-compartment models can involve hundreds of compartments with distinct ion channel complements [65]. Second, ion channel densities are known to vary non-uniformly along the dendritic tree [66], and neuromodulators can act in a compartment-specific manner. For instance, Williams et al. [67] recently showed that neuropeptide neuromodulation differentially controls dendritic potassium channels depending on whether the dendrite bears an axon or not, and Mäki-Marttunen and Mäki-Marttunen [68] demonstrated computationally that HCN channel modulation has opposing effects at proximal versus distal dendritic compartments. These observations suggest that a single neuromodulation controller would be insufficient in spatially extended neurons, and that independent or hierarchical controllers for distinct compartments may be required.

We have added a paragraph in the Discussion to outline these points and identify possible directions for future work.

Changes in manuscript: *(Discussion, new paragraph)* Added: "The controlled neuromodulation framework presented here operates on single-compartment models. Extending it to multi-compartment models with dendritic conductances is a natural direction for future work. In principle, the DIC framework generalizes to spatially extended models, as the sensitivity matrix can be augmented to include dendritic conductances and electrotonic coupling terms [65]. However, this extension raises challenges related to the increased dimensionality of the conductance space

and the established non-uniformity of dendritic ion channel distributions [66]. Furthermore, recent experimental evidence demonstrates that neuromodulators can act in a compartment-specific manner [67], and computational studies show that the same neuromodulatory action can have opposing effects at different dendritic locations [68]. These observations suggest that spatially distributed or hierarchical control architectures may be required. Addressing these challenges in the context of controlled neuromodulation remains an open direction for future work."

Response to Reviewer 3

We thank Reviewer 3 for their thorough and detailed review. Their constructive comments on both the scientific content and the presentation of the figures were extremely helpful in improving the quality of the manuscript. We appreciate their recognition of our framework and methodology, and we believe that addressing their feedback has substantially strengthened the paper.

Major comments

R3.1 *"Ih Currents: It is unclear as to why the neuromodulatory response, as the authors have modelled, instantly changes two ion channel types (A-type K and Slow Ca type) and not the Ih-channels. Ih-current carrying ion channels are well known to be sensitive to cAMP levels: When cAMP binds to the channel, it shifts the activation curve of the channel to more positive voltages. As a result, the channel opens faster and at less negative voltages. Given that the Ih currents set the pacemaker currents in the rhythmic nature of the spiking, it seems natural to include the instantaneous changes to these channels in this context. Please justify this omission or include this in your study."*

We thank the Reviewer for this important point. The Reviewer is correct that Ih channels are well-established targets of neuromodulation through cAMP-dependent pathways [48], and their omission from the main analysis required justification. Our choice to modulate $\bar{g}_{\mathrm{A}}$ and $\bar{g}_{\mathrm{CaS}}$ in the main figures was motivated by the fact that these two conductances provide the most direct and interpretable control over the spiking-to-bursting transition in the STG model, as established in our previous work [30, 33]. However, the controlled neuromodulation framework is agnostic to the specific subset of modulated channels, and we agree that demonstrating this generality is important.

In response to this comment, we have conducted additional experiments in which H-type channels ($\bar{g}_{\mathrm{H}}$) are included as a neuromodulatory target alongside $\bar{g}_{\mathrm{CaS}}$. These experiments, presented in Supporting Information, S1 Appendix, reproduce the main results of Fig 2 with neuromodulation acting on H-type channels. The results are qualitatively identical: sharp neuromodulation combined with calcium homeostasis

leads to unreliable outcomes, whereas controlled neuromodulation produces robust and reliable modulation. This confirms that our findings are not specific to the particular subset of modulated channels but rather emerge from the activity-dependent nature of the control action itself.

We have added a paragraph in the main text (at the end of the third Results subsection) directing the reader to these supplementary results and explicitly acknowledging the sensitivity of Ih channels to cAMP.

Changes in manuscript:

- *(Results, third subsection)* Added: "The results above were obtained with controlled neuromodulation acting on A-type potassium ($\bar{g}_{\mathrm{A}}$) and slow calcium ($\bar{g}_{\mathrm{CaS}}$) conductances. However, because the neuromodulation controller is agnostic to the specific neuromodulator and signaling cascade, we also conducted experiments in which H-type channels ($\bar{g}_{\mathrm{H}}$) are modulated, given that these channels are well known to be sensitive to cAMP levels [48]. An analogous figure to Fig 2, with neuromodulation acting on H-type channels, can be found in Supporting Information, S1 Appendix. These experiments yield similar results: failure under sharp neuromodulation and robust function under controlled neuromodulation. This confirms that the observations are not specific to the particular subset of modulated channels, but rather emerge from the activity-dependent control action itself."
- *(S1 Appendix)* Added supplementary figure reproducing Fig 2 with neuromodulation acting on H-type channels.

R3.2 *"Temporal dynamics: Why is the neuromodulatory perturbation not relaxing after a while? [nmod] is presented, and a change of a dynamical interaction is seen, but this assumes that the [nmod] persists for long – necessarily longer than the two homeostatic mechanisms to kick in. If the neuromodulator doesn't wash out, the homeostasis isn't responding to a perturbation; it's responding to a permanent change in the environment. Most neuromodulators would have some adaptation even. One would anticipate that the A-type conductivity and the CaS would return naturally to their previous values once the neuromodulation is washed away. Please justify why this is modelled this way, or include this in your study."*

We thank the Reviewer for this comment, which raises an important point about the temporal dynamics of neuromodulatory signals.

The sustained nature of the neuromodulatory input in our simulations reflects the biological context we aim to model. Many neuromodulatory states in biological systems are maintained over extended periods by tonic release from projection neurons [46, 47]. A well-characterized example is the gastric mill rhythm in the STG,

which is driven by continuous neuromodulatory input from descending modulatory neurons and persists only as long as this input is maintained [47]. In this context, the calcium-homeostatic controller is indeed responding to a sustained change in the neuromodulatory environment rather than to a transient perturbation, and the relevant question is whether both controllers can coexist under this sustained regime.

That said, we agree that examining the washout scenario is informative and provides additional insight into the robustness of the framework. We have therefore added a washout analysis in the revised manuscript and in Supporting Information, S1 Appendix. We examined two cases: washout before calcium-homeostatic compensation has converged (at t_{after}) and washout after convergence (at $t_{\text{compensated}}$).

In both cases, tonic spiking is recovered across the population under both sharp and controlled neuromodulation, though for fundamentally different reasons and with different levels of fidelity. Under sharp neuromodulation, calcium-homeostatic compensation has been driving conductances along the native tonic spiking scaling direction throughout, so that tonic spiking is the only remaining stable activity pattern; recovery upon washout is therefore expected, as the system has no other stable attractor. However, at $t_{\text{compensated}}$, the conductance distribution after washout does not exactly match its pre-neuromodulation state: because calcium homeostasis has been compensating for the sharp perturbation, the resulting conductance profiles are subtly altered, yielding a slightly different firing frequency distribution. This quantitative drift suggests that repeated cycles of sharp neuromodulation and washout could progressively degrade neuronal function. Under controlled neuromodulation, the neuromodulatory reference is removed upon washout, and the calcium-homeostatic controller regains sole authority over conductance tuning, returning the system to tonic spiking. Because the two controllers operate in coordination rather than in competition, the pre-neuromodulation state is faithfully recovered.

Changes in manuscript:

- *(Results, second subsection)* Added a paragraph on washout dynamics: "The differences between sharp and controlled neuromodulation can also be highlighted through neuromodulator washout... At both t_{after} and $t_{\text{compensated}}$, washout recovers tonic spiking across the population under both sharp and controlled neuromodulation, though for fundamentally different reasons and with different levels of fidelity..."
- *(S1 Appendix)* Added washout figures for both sharp and controlled neuromodulation at t_{after} and $t_{\text{compensated}}$.

Minor comments

R3.m1 *"Overall, all the figures could be much better."*

We agree and have substantially revised all figures in the manuscript. The main improvements include: addition of proper axis tick marks and labels throughout; inclusion of single-neuron representative traces and population activity distributions (Fig 2C and Fig 2F); separation of sharp and controlled neuromodulation outcomes into distinct panels (Fig 3C-D); clearer arrow legends in the schematic figures (Fig 4); explicit y-axis labels on all violin plots (Figs 3, 5); a dedicated panel for initial conductance values in the network figure (Fig 6B); and revised schematics for the homeostatic and neuromodulatory cascades (Fig 1), created in BioRender. We address the specific figure comments in detail below (R3.m2–R3.m7).

R3.m2 *"Figure 1: An equivalent to Fig. 2A could be placed here. The top row (unlabeled, uncaptioned) represents three exemplar traces from the population at three different time points (unlabeled, not mentioned, correspond to dashed vertical lines?). Middle row, consider showing the trace of one neuron and showing the rest as histograms of population at the vertical dashed lines. [nmod] is a notation that is only accessible after reading the methods. Please consider a better label for the perturbation. Include the vertical dashed lines in the bottom row for the ca-conc."*

We have substantially restructured the presentation of the simulation results in response to this and related comments. In the revised manuscript, the sharp neuromodulation results (originally Fig 1) are now presented as panels A–C of Fig 2. Panel A shows the population conductance trajectories (as before, but with improved axis labels and tick marks). Panel B shows the mean intracellular calcium concentration over time, now with vertical dashed lines indicating the three key time points (t_{before}, t_{after}, $t_{\text{compensated}}$). Panel C combines the Reviewer's suggestions: the top row shows three representative single-neuron voltage traces at each time point, while the bottom row shows the population activity distributions as violin plots. We have also replaced the "[nmod]" notation with a descriptive label ("neuromodulator") in the figure (dashed blue line indicating the onset of neuromodulation). The calcium homeostasis schematic (formerly part of original Fig 2A) is now presented in Fig 1A, which precedes all simulation results.

Changes in manuscript:

- *(Fig 2, panels A–C; formerly Fig 1)* Restructured into panels A (conductance trajectories), B (calcium dynamics with vertical dashed lines at key time points), and C (representative single-neuron traces and population activity distributions). Replaced "[nmod]" label with "neuromodulator".

R3.m3 *"Figure 2: Same as for Figure 1. The schematic could be improved."*

The original Fig 2 has been split and reorganized as part of the restructuring. Fig 1 now contains improved schematics of the calcium-homeostatic cascade (panel A) and of both sharp and controlled neuromodulation (panel B). The controlled neuromodulation schematic now emphasizes the activity-dependent feedback loop and its relationship to the calcium-homeostatic loop. The controlled neuromodulation simulation results that were previously in the original Fig 2 are now presented in Fig 2 panels D–F, which follow the same three-panel structure as panels A–C (conductance trajectories, calcium dynamics, and representative traces with population distributions).

Changes in manuscript:

- *(Fig 1; formerly Fig 2 schematics)* Now dedicated to schematics only: panel A for calcium homeostasis, panel B for sharp (left) and controlled (right) neuromodulation. Created in BioRender [32].
- *(Fig 2, panels D–F; formerly Fig 3 in previous revision)* Showing controlled neuromodulation results, with the same panel structure as panels A–C.

R3.m4 *"Figure 3: Perhaps indicate the time points from Figure 1, 2 here for the A, B. Consider showing the trajectories for fewer neurons, and show the conductivity of the population as a histogram of before and after on the x and y axis. Subplot C, has the inset plot Burstiness (Hz) [y-axis] versus X-axis (is it time?): this is totally unclear what you wish to show here. I realize that 65% implies that 65% of the neuron models are bursting (what happens to the rest) at the end. But what is the raster (raster-like) plot supposed to show? The labels "Initial condition from previous step" and "final condition from current step" could be improved."*

The main changes of Fig 3 are as follows. Panels A and B show the conductance-space trajectories during tonic spiking calcium homeostasis and during neuromodulation, respectively, with clearer marker labels (white circles for initial conditions, green triangles for final conditions). The former panel C has been split into two separate panels: panel C for sharp neuromodulation and panel D for controlled neuromodulation, each now including a violin plot showing the population burstiness distribution at the end of the compensation phase. The burstiness axis is labeled in Hz^2 on a linear scale, and the time points are indicated consistently with the t_{before}, t_{after}, and $t_{\text{compensated}}$ notation used throughout the manuscript. We have also replaced the labels "Initial condition from previous step" and "Final condition from current step" with the more descriptive markers and time-point labels.

Changes in manuscript:

- *(Fig 3)* Split panel C into panels C (sharp) and D (controlled), each with violin plots of population burstiness in Hz^2. Improved all marker and axis labels.

Adopted consistent time-point notation (t_{before}, t_{after}, and $t_{\text{compensated}}$).

R3.m5 *"Figure 4: While I recognize the value of the insight from this schematic, I fear it is misrepresenting the sharp neuromodulation (subplot A). Based on Figure 1 bottom row, the Ca-levels are restored to the target Ca level. Please clarify this. In A, what is the dashed arrow representing? The labels could be better. There is too much text. Could this plot be generated from the actual simulations instead?"*

We thank the Reviewer for this careful observation. The Reviewer is correct that under sharp neuromodulation, calcium levels are eventually restored to the target (as shown in Fig 2B). The original schematic could have been misread as suggesting that calcium homeostasis fails entirely, which is not the case. What fails is the preservation of the neuromodulated firing pattern: calcium returns to its target, but the neuron drifts away from the bursting isocline in the process. We have revised the schematic (Fig 4) to make this distinction explicit. Both panels A and B now show the neuron reaching the target calcium isocline at $t_{\text{compensated}}$, but in panel A the final state (red star) lies off the bursting isocline, whereas in panel B it lies at the intersection of both isoclines.

Regarding the dashed arrow, it represents the direction of calcium-homeostatic compensation when the neuron is in tonic firing mode (*i.e.*, the native scaling direction). We have added an explicit legend at the bottom of the caption.

Regarding generating the plot from actual simulations: the schematic is intentionally simplified to convey the geometric intuition of the mechanism in a more general way. The actual simulation trajectories are shown in Fig 3C-D, which serves as the data-based counterpart. We believe that keeping both representations (schematic for intuition, simulation for evidence) best serves the reader.

Changes in manuscript:

- *(Fig 4)* Revised the schematic so that the final state under sharp neuromodulation ($t_{\text{compensated}}$, red star) is shown on the target calcium isocline but off the bursting isocline, accurately reflecting that calcium is restored but the firing pattern is not. Added consistent t_{before}, t_{after}, $t_{\text{compensated}}$ labels and an explicit arrow legend.

R3.m6 *"Figure 5: The raster (raster-like) plots are unclear. What is the X-axis? What is the Y-axis? Frequency of burstiness? There is redundant labeling "Control condition", "After blockage", "With compensation"."*

We have revised Fig 5 to address these concerns. The activity distribution plots are now presented as violin plots with explicitly labeled axes: the y-axis shows burstiness in Hz^2 on a linear scale, and each violin corresponds to a distinct condition indicated

by x-axis tick labels (“Control condition”, “After blockade”, “With compensation”). We have removed the redundant text labels that previously appeared above the plots, as this information is now conveyed by the x-axis ticks. The caption specifies that a burstiness of zero indicates the absence of multi-spike bursting.

Changes in manuscript:

- *(Fig 5)* Replaced raster-like plots with violin plots with explicit axis labels (burstiness in Hz^2). Moved condition labels (“Control condition”, “After blockade”, “With compensation”) to x-axis ticks, removing redundant text annotations.

R3.m7 *“Figure 6: Subplot A, right side: what do the colors here mean? There are some interesting results here, but they are rushed. Please consider elaborating on these.”*

In the original Fig 6A right (now Fig 6B), the colors correspond to the five neurons of the network (N1–N5), with each neuron color-coded consistently with the network schematic. The bar chart shows the initial conductance values for each neuron, with multiplier coefficients applied to all conductances to facilitate visualization in a non-logarithmic graph, following the convention of [43]. We have revised the figure to make this explicit in the caption and have added a color legend.

Regarding the request for elaboration, we have added a substantial mechanistic paragraph in the Results section that explains why sharp neuromodulation fails and controlled neuromodulation succeeds at the network level. The explanation traces the mechanism back to the single-neuron results: under sharp neuromodulation, calcium homeostasis pulls slow-rhythm neurons off the bursting isocline, and since the half-center oscillator requires both neurons to maintain robust bursting, the loss of bursting in even a single neuron collapses the slow rhythm. Under controlled neuromodulation, the activity-dependent feedback keeps each neuron on its target isocline. We also discuss the effect on the fast rhythm and its consistency with experimental observations.

Changes in manuscript:

- *(Fig 6)* Added color legend in panel B. Improved caption to explain multiplier coefficients and color coding.
- *(Results, fifth subsection)* Added a detailed mechanistic paragraph beginning with: “More specifically, under sharp neuromodulation combined with calcium homeostasis (Fig 6C middle), the slow rhythm initially emerges but rapidly deteriorates…”

R3.m8 *“Please consider taking the section on DA neuron out of the main manuscript entirely. It feels out of place. Perhaps you can consider writing it as another case study of this mechanism and mention it only in the discussion.”*

We agree with the Reviewer that the DA neuron paragraph interrupted the flow of the Results section, where the focus is on the STG model. We have moved the DA neuron case study out of the main Results and into the Discussion, where it now serves as additional evidence for the generality of the framework.

Changes in manuscript:

- *(Results)* Removed the paragraph beginning with "To demonstrate the generality of this approach, we applied the same framework to a midbrain dopaminergic neuron model...".
- *(Discussion)* Added the DA neuron case study as a new paragraph: "To further demonstrate the generality of the proposed framework, we applied it to a midbrain dopaminergic neuron model adapted from [23]. As in the STG model, a transition from tonic spiking (pacemaking) to strong bursting was induced by modulating N-type and L-type calcium conductances ($\bar{g}_{\mathrm{CaN}}$ and $\bar{g}_{\mathrm{CaL}}$) through controlled neuromodulation. Calcium-homeostatic compensation continuously regulated calcium levels, ensuring a reliable and robust outcome despite initial conductance degeneracy within the neuronal population (see Supporting Information, S1 Appendix, for details)."

R3.m9 *"Throughout the work, "homeostatic controller" is used. I urge you to be specific about it and call it a calcium-homeostatic controller all through. There can be many other homeostatic controllers; in your work you re-implemented O'Leary's Ca set-point homeostatic controller. In one way, your work is also a homeostatic controller that combines Ca concentration and neuromodulatory response."*

We fully agree with the Reviewer that the term "homeostatic controller" is overly generic and potentially ambiguous, given the diversity of homeostatic mechanisms in biology. Following this suggestion, we have systematically replaced "homeostatic controller" with "calcium-homeostatic controller" (or "calcium homeostasis" where appropriate) throughout the manuscript, to clearly identify the specific mechanism we model, namely the calcium set-point controller from O'Leary et al. (2014). We have also added a clarifying sentence at first use in the Introduction to distinguish this specific mechanism from broader homeostatic processes.

Changes in manuscript:

- Throughout the manuscript, all occurrences of "homeostatic controller" have been replaced with "calcium-homeostatic controller", and "homeostatic compensation/regulation" with "calcium-homeostatic compensation/regulation".
- *(Introduction)* Added at first use: "We model intrinsic homeostasis using the calcium-homeostatic controller proposed by [29], which adjusts all ionic con-

ductances based on the deviation of the neuron average intracellular calcium level from a target set-point. We refer to this mechanism as *calcium homeostasis* throughout this work, to distinguish it from other forms of homeostatic plasticity."

- *(Materials and methods, "The homeostatic controller" subsection)* Section title changed to: "The calcium-homeostatic controller".

R3.m10 *"Please consider changing the names of what you call [nmod] followed by [sharp neuromodulatory controller] versus an [interacting neuromodulatory controller]. These notions are difficult to follow. You could revise the naming conventions to better reflect on what your modelling effort is trying to achieve. Perhaps considering your work as a nested feedback loop, that is over the ca-homeostasis would be easier to interpret."*

We agree that the original naming conventions were unclear. In the revised manuscript, we have adopted the following terminology consistently:

- **Sharp neuromodulation**: a direct, externally driven, activity-independent modification of ion channel conductances. This term emphasizes the instantaneous and open-loop nature of the intervention.
- **Controlled neuromodulation**: an activity-dependent feedback mechanism that adjusts ion channel conductances through an intracellular signaling cascade. This term emphasizes the closed-loop, adaptive nature of the mechanism.
- **Calcium homeostasis** (or **calcium-homeostatic controller**): the calcium set-point regulation mechanism from [29].

The "[nmod]" notation has been replaced throughout the figures with the descriptive label "neuromodulator". In the equations in the Materials and Methods section, [nmod] is retained as the mathematical symbol for neuromodulator concentration but is now introduced with a clear definition at first use.

We considered the Reviewer's suggestion of framing controlled neuromodulation as a nested feedback loop over calcium homeostasis. While we appreciate the insight, the controlled neuromodulation loop does not strictly act over the calcium-homeostatic loop: rather, it forms a separate feedback loop over the neuronal activity itself. Both loops act in parallel on the same set of conductances, but with different sensed variables (neuronal activity for neuromodulation, intracellular calcium for homeostasis) and different targets. The revised Fig 1B schematic now visually represents this architecture, showing the two feedback loops acting in parallel on the neuron, with controlled neuromodulation sensing neuronal activity and calcium homeostasis sensing intracellular calcium.

Changes in manuscript:

- Throughout the manuscript, consistently applied the terminology "sharp neuromodulation" and "controlled neuromodulation", with clear definitions at first use in both the Introduction and the Results.
- *(All figures)* Replaced "[nmod]" notation with the descriptive label "neuromodulator".
- *(Fig 1B)* Revised schematic to visually represent the two parallel feedback loops: controlled neuromodulation sensing neuronal activity, and calcium homeostasis sensing intracellular calcium, both acting on the same set of conductances.
- *(Results, first subsection)* Added to the definition of sharp neuromodulation: "applied independently of neuronal activity".

R3.m11 *"Please change the title to better reflect what the paper is showcasing. Something in the lines of "Interplay between calcium-mediated homeostasis and neuromodulatory control in STG neurons guarantees robustness"."*

We thank the Reviewer for this suggestion. We agree that the original title did not sufficiently convey the specific mechanisms at play. We have revised the title to: *"Activity-dependent neuromodulation and calcium homeostasis cooperate to produce robust and modulable neuronal function."* This revised title makes explicit that the homeostatic mechanism is calcium-based (consistent with the terminology changes described in R3.m9) and highlights the activity-dependent nature of controlled neuromodulation, which is the key property distinguishing it from sharp neuromodulation. We chose not to restrict the title to STG neurons, as the framework is also demonstrated on midbrain dopaminergic neurons and central pattern generator networks.

Changes in manuscript: *(Title)* Changed from "Controlled neuromodulation naturally interacts with homeostasis to produce robust and modulable neuronal function" to "Activity-dependent neuromodulation and calcium homeostasis cooperate to produce robust and modulable neuronal function".
